# Supplementary material for: Research on adaptive hydraulic drive optimization control of concrete mixing tank truck for open-pit mine
Source: PLoS One. 2024 Oct 9;19(10):e0310249. doi: 10.1371/journal.pone.0310249 (PMC11463772; doi:10.1371/journal.pone.0310249)
Supplement: S1 File — (DOCX) [file pone.0310249.s001.docx]

Minimal Data statement

All relevant data are within the manuscript and its Supporting Information files.All the minimum data sets appearing in the manuscript come from the Internet, materials within the research group, and contacts between corresponding companies. We have uploaded all the data sets in the Major Revision, and the Final Figure File is also uploaded in the Current Task Assignments.
