# Supplementary material for: Research on adaptive hydraulic drive optimization control of concrete mixing tank truck for open-pit mine
Source: PLoS One. 2024 Oct 9;19(10):e0310249. doi: 10.1371/journal.pone.0310249 (PMC11463772; doi:10.1371/journal.pone.0310249)
Supplement: Attachment — Submitted filename: PONE-D-24-25486.docx [file pone.0310249.s003.docx]

Dear Authors here is the comments for the manuscript as follows:

ABSTRACT:

Your review of the abstract provides a thorough analysis of its content, structure, and effectiveness in conveying the key aspects of the research. Let's organize the review into a more structured format to enhance clarity and readability.

Title: Abstract Review

Language and Consistency:

The abstract is well-written and free of any major language errors. The flow of ideas is logical, and the content is consistent with the title and the expected structure of an abstract. However, there are a few minor suggestions for improvement:

1. Clarify the referent of "it" in the sentence "Then, it analyzes its hydraulic drive control characteristics and structural applications" (presumably referring to the proposed method).

2. Rephrase the sentence "Under filling rates of 1.5 and 2.0, two modes of step steering and double lane change are simulated, respectively" to improve clarity, such as "Step steering and double lane change modes are simulated under filling rates of 1.5 and 2.0, respectively."

Recommendations:

1. Clarify the referent of "it" in the sentence discussing the analysis of hydraulic drive control characteristics and structural applications.

2. Rephrase the sentence about the simulation of step steering and double lane change modes under different filling rates to improve clarity.

INTRODUCTION:

The authors highlight the challenging environmental conditions in open-pit mines and the safety issues faced by mining concrete mixing tank trucks, particularly the non-axisymmetric problems caused by fluid sloshing in the tank and its impact on vehicle dynamics when coupled with excitations from complex road surfaces.

there are several areas where the introduction could be improved:

1. Insufficient background information: While the authors describe the general conditions in open-pit mines, they do not provide a clear connection between these conditions and the specific problem of fluid sloshing in concrete mixing tank trucks. They should explicitly state how these conditions contribute to the fluid sloshing problem or make it more challenging to address.

2. Limited discussion of existing research: The introduction would benefit from a more comprehensive review of existing literature on the topic. The authors briefly mention potential solutions, such as advanced suspension systems, intelligent transmission systems, and remote diagnosis systems, but do not provide specific examples or references to support their claims. They also state that these solutions are insufficient without a clear explanation of why they are inadequate. A more detailed discussion of existing solutions and their limitations, supported by relevant references, would help readers assess the novelty and significance of the proposed research.

3. Lack of clarity in research objectives: The authors state that their main objective is to analyze the stress conditions of mining concrete mixing tank trucks and optimize the control performance to improve driving stability, high-speed accuracy, and robustness. However, this statement is not clearly connected to the identified problem of fluid sloshing and its impact on vehicle dynamics. The authors should revise this statement to clearly articulate how their research objectives address the limitations of existing solutions and the specific problems they have identified.

4. Inconsistency in problem description: There is an inconsistency between the abstract and the introduction regarding the cause of the non-axisymmetric problem. In the abstract, the authors mention that the non-axisymmetric problem is caused by fluid sloshing in the tank during the driving process, while in the introduction, they state that the fluid sloshing is affected by the excitation of the complex road surface, which makes the fluid sloshing coupled with the body dynamics. This inconsistency should be clarified to ensure a clear understanding of the problem.

5. Insufficient justification for the proposed method: The authors introduce their proposed method, the firefly filter compensation optimization adaptive control algorithm with an elastic mechanism, without providing sufficient background information or justification. They should include a brief explanation of these concepts, their advantages over other potential approaches, and why they are suitable for addressing the hydraulic drive control problem in mining concrete mixing tank trucks. Additionally, the references cited in this section are not properly introduced or discussed, making it difficult for readers to understand their relevance to the current study.

6. Lack of research questions or hypotheses: The introduction does not explicitly state the research questions or hypotheses that the study aims to address. Clearly defining these would help readers understand the specific goals and expectations of the research.

A deeper analysis of the introduction by quoting specific passages and providing a critical assessment.

Quote 1: *"The mining site of an open-pit mine is usually vast, with an open working surface and complex environmental conditions in the mining area. It often faces harsh climatic conditions, such as high temperatures, cold, wind, and sand. In addition, the working range of an open-pit mine is extensive, the working surface often changes, the road conditions are relatively complex, the transportation distance is long, and the transportation task is heavy [1-6]."*

comment: This passage sets the context for the research problem by describing the challenging conditions in open-pit mines. However, the authors do not provide a clear connection between these conditions and the specific problem of fluid sloshing in concrete mixing tank trucks. They could improve this by explicitly stating how these conditions contribute to the fluid sloshing problem or make it more challenging to address.

Quote 2: *"For such problems, they can be solved by structural improvement or control method optimization. There are adjustable advanced suspension systems, intelligent transmission systems, and remote diagnosis systems that can adapt to changing terrain conditions and perform excellently in passing obstacles and slopes. However, the above solutions are still insufficient to solve the driving problems of mining concrete mixing tank trucks in complex environments such as uphill and sloped roads."*

comment: The authors mention potential solutions, such as structural improvements and control method optimization, but they do not provide specific examples or references to support their claims. They also state that existing solutions are insufficient without providing a clear explanation of why they are inadequate. This lack of detail makes it difficult for readers to assess the novelty and significance of the proposed research.

Quote 3: *"Therefore, analyzing the stress conditions of mining concrete mixing tank trucks and optimizing the control performance effect [8-10]."*

Comment: This sentence is incomplete and lacks clarity. The authors should revise it to clearly state the objective of their research and how it addresses the limitations of existing solutions. Additionally, the references [8-10] are not properly introduced or discussed, making it difficult for readers to understand their relevance to the current study.

Quote 4: *"In order to improve the hydraulic drive control of a concrete mixer transport tank truck for mining, this paper adopts the firefly filter compensation optimization adaptive control algorithm [16-19] and introduces an elastic mechanism [20] to speed up the optimization process."*

Comment: The authors introduce their proposed method without providing sufficient background information on the firefly filter compensation optimization adaptive control algorithm and the elastic mechanism. They should include a brief explanation of these concepts and why they are suitable for addressing the hydraulic drive control problem in mining concrete mixing tank trucks. Furthermore, the references [16-20] are not properly discussed, and their relevance to the current study is not clearly established.

The authors should address the identified inconsistencies, provide a clearer problem statement, and better justify their proposed approach. They should also include a more comprehensive review of existing literature, clearly define their research objectives and hypotheses, and establish a stronger connection between the challenging conditions in open-pit mines and the specific problem of fluid sloshing in concrete mixing tank trucks. By addressing these issues, the authors can strengthen the introduction and provide a more solid foundation for the rest of the paper.

REVIEW OF SECTION 2.1:

The authors aim to study the motion state of a mining concrete mixing and transport tank truck driving on a sloped platform by conducting a force analysis of the vehicle body.

Quote 1: "In order to study the motion state of a mining concrete mixing and transport tank truck when it is driving on a platform with a slope angle, the force analysis of the vehicle body is carried out, as shown in Fig. 1, where L₁,L₂ the distances along the slope direction between the center of mass of the concrete tank truck and the contact points between the front and rear tires and the road slope, respectively, and H is the average distance between the center of mass and the slope."

Comment: The authors clearly describe the purpose of this section and introduce the variables L₁, L₂, and H, which are used in the force analysis of the vehicle body on a sloped platform. The explanation is concise and easy to understand. Therefore, no need to fix anything.

Quote 2: "According to the static equilibrium equation, we can get: Ft1 + Ft2 + Ft3 = mgsinθ (1) Fn1 + Fn2 + Fn3 = mgcosθ (2)"

Comment: Equations (1) and (2) represent the static equilibrium equations for the forces acting on the vehicle body in the tangential and normal directions. The equations are consistent with the force diagram shown in Fig. 1. HOWEVER, the authors introduce Ft3 and Fn3 without prior definition, which may confuse readers. It would be better to clarify that these forces represent the tangential and normal forces acting on the third axle of the vehicle.

Quote 3: "The mining concrete mixing transport tanker is driving on the slope platform, and the interaction between the wheels and the slope is shown in Fig. 2."

Comment: The authors introduce a new force interaction model between the wheels and the slope platform, which is essential for understanding the vehicle's dynamics on the slope. The description is clear and supported by a visual representation in Fig. 2.

Equations (3) to (8):

Comment: Equations (3) to (8) describe various force interactions and parameters related to the wheel-slope interaction, such as the approach angle, maximum stress point approach angle, normal stress, and shear stress. The equations are derived from well-established models, such as the Bekker model for terrain-vehicle interaction. The authors have provided clear explanations for each equation and the variables involved.

HOWEVER, THERE ARE A FEW INCONSISTENCIES AND AREAS THAT REQUIRE FURTHER CLARIFICATION:

1. In Equation (6), the shear stress τ is expressed as a function of the normal stress σ, but σ is not included in the equation. The authors should clarify this inconsistency and ensure that all variables are properly defined.

2. In Equation (7), the authors use θ₁ and θₘ without prior definition. They should clearly explain the meaning of these variables to avoid confusion and improve the equation's readability.

3. The authors introduce the tire sinkage model in Equation (9) but do not provide a clear explanation of how this model relates to the overall mechanical model of the slope platform. They should clarify the significance of the tire sinkage model and its role in the analysis.

4. The tire sinkage model in Equation (9) includes several parameters, such as b, kc, and kφ, which are not defined in the text. The authors should provide definitions and explanations for these parameters to ensure that readers can fully understand the equation.

5. In Equation (10), the authors introduce the sinking coefficient λ to represent the relationship between the front and rear wheel sinkage. However, they do not provide a clear explanation of how this coefficient is determined or its physical significance in the context of the mining concrete mixing transport tanker. Additional information on the sinking coefficient would improve the reader's understanding of the mechanical model.

REVIEW OF SECTION 2.2:

The authors describe the closed hydraulic drive system used in most mining concrete mixing and transport tank trucks, supported by a schematic representation in Fig. 3. The explanation of the system's components and their functions is clear and concise, allowing readers to understand the system's overall structure and operation.

Quote 1: "Most mining concrete mixing and transport tank trucks use a closed hydraulic drive system, the structure of which is shown in Fig. 3. The oil ports at both ends of the variable pump are connected end to end with the oil inlet and return ports of the quantitative motor to form a closed loop."

Comment: The description of the closed hydraulic drive system is clear and supported by Fig. 3, which enhances the reader's understanding of the system's layout.

Quote 2: "The engine drives the variable pump to provide high-pressure oil to the system, and the variable mechanism can be adjusted according to the control signal to achieve the speed and steering control of the quantitative motor. The system oil replenishment circuit consists of a one-way valve, an accumulator, an oil replenishment pump, an oil tank, and a flow valve to replenish the leaked oil in time for the system to prevent cavitation."

Comment: The explanation of the system's functioning is concise and informative, covering the roles of key components such as the variable pump, variable mechanism, and oil replenishment circuit.

Equations (13) to (16):

Comment: The equations presented in this section are consistent with the fundamental principles of fluid mechanics, rotational dynamics, and gear ratios.

HOWEVER, there are some inconsistencies and areas that require further clarification:

1. Units: The authors do not provide units for the variables used in the equations, which may lead to confusion and difficulty in reproducing the results. They should include the appropriate units for each variable to ensure clarity and reproducibility.

2. Assumptions and limitations: The authors do not discuss the assumptions or limitations (try to refer to previous works) of the hydraulic drive system model. They should acknowledge any simplifications or approximations made in deriving the equations and discuss their potential impact on the model's accuracy. This would help readers understand the model's applicability and limitations.

3. Load torque: In Equation (15), the load torque TL is not clearly defined or explained. The authors should provide more information on how this torque is determined and what factors influence its value. This would enable readers to better understand the torque balance equation and its implications for the hydraulic drive system.

4. Gear ratio: In Equation (16), the authors should specify whether the gear ratio iv is the overall gear ratio or the ratio of a specific gear pair in the transmission. Clarifying this would help readers interpret the relationship between motor speed and vehicle speed more accurately.

5. Bulk modulus: In Equation (14), the authors use the term "effective bulk modulus" (βe) without providing a clear explanation of what it represents or how it is determined. They should offer a brief description of the bulk modulus and its significance in the flow continuity equation.

Your critical analysis of section 3 "Design of adaptive PID controller based on elastic FA algorithm optimization filter" is comprehensive and well-structured. You have examined the content, equations, and algorithms, providing insights into the consistency, scientific accuracy, and clarity of the presented material. Let's build upon your analysis and address the inconsistencies and areas that require further clarification.

REVIEW OF SECTION 3:

The authors propose an adaptive PID controller based on the elastic FA algorithm optimization filter to overcome the limitations of traditional PID control in handling the complex driving conditions of mining concrete mixing tank trucks. They provide a clear explanation of the motivation behind their proposed approach and its potential benefits.

SECTION 3.1: OPTIMIZATION PRINCIPLE OF THE ELASTIC FA ALGORITHM

The authors describe the optimization principle of the elastic FA algorithm, including the gravity and movement mechanisms, elasticity optimization strength control, and particle density detection. The explanations of the concepts and equations involved, such as particle fluorescence brightness, particle attraction, and particle position update, are detailed and consistent with the principles of the FA algorithm and the elastic mechanism.

HOWEVER, THERE ARE A FEW INCONSISTENCIES AND AREAS THAT REQUIRE FURTHER CLARIFICATION:

1. Equation (23): The authors should clarify whether the variable t represents time or iteration number and ensure consistency throughout the section.

2. Cutoff condition: The authors should provide a clear definition and explanation of the "cutoff condition" in the context of elasticity optimization strength control and how it relates to the optimization process.

3. Equation (32): The authors should offer guidance on selecting an appropriate value for the constant c and discuss its impact on the optimization process.

REVIEW OF SECTION 3.1.1:

The authors introduce three key concepts in this section: particle fluorescence brightness, particle attraction, and particle position update. They provide equations and explanations for each concept, which are generally clear and consistent with the principles of the FA algorithm.

Particle Fluorescence Brightness:

Equation (23) defines the particle fluorescence brightness Iⁿᵢ(t) as a measure of the particle's position, with higher values indicating better positions. The equation is consistent with the concept of fluorescence brightness in the FA algorithm.

Comment: The authors use inconsistent notation for the particle index, denoting it as "i" in Equation (23) and "ρ" in subsequent equations and explanations. They should maintain consistent notation throughout the section to avoid confusion.

Particle Attraction:

Equation (25) defines the attraction of the best particle ρmax to particle ρ, which is consistent with the concept of attraction in the FA algorithm.

Comment: The authors use inconsistent notation for the distance between particles, denoting it as "rᵢmax(t)" in Equation (25) and referring to it as "rⁿᵢmax(t)" in the explanation. They should clarify the correct notation and ensure consistency throughout the section.

Particle Position Update:

Equation (26) describes the process of updating particle positions based on the attraction of the best particle and a random component, which is consistent with the concept of particle movement in the FA algorithm.

Areas requiring further clarification:

1. Gain coefficients: The authors should provide guidance on selecting appropriate values for the gain coefficients α₁ and α₂ and discuss their impact on the optimization process.

2. Random function: The authors should clarify the range of the uniform distribution satisfied by the random function "rand" to ensure reproducibility.

3. Convergence properties and termination criteria: The authors should provide insight into how the algorithm determines when to stop updating particle positions and consider the solution converged.

REVIEW OF SECTION 3.1.2:

The authors introduce the concept of elasticity optimization strength control to prevent unreasonable particle aggregation and over-optimization, which can lead to computational burden and low-quality convergence. They propose using the proportion of high-likelihood particles, the maximum number of iterations, and a particle density detection mechanism to control the optimization intensity.

Particle Density:

Equation (27) defines particle density ρcr(m) as the number of particles within a specified judgment radius rjudg around the optimal particle ρmax(n). The equation is consistent with the concept of particle density and provides a quantitative measure of particle aggregation.

Comment: The authors use inconsistent notation for the particle index, denoting it as "i" in Equation (27) and "ρ" in the previous section. They should maintain consistent notation throughout the paper to avoid confusion.

Ratio of High-Probability Particles:

Equation (28) defines the ratio of high-likelihood particles pr(m) as the ratio of the particle density ρcr(m) to the total number of particles N. The equation is consistent with the concept of measuring the proportion of particles that are likely to be close to the optimal solution.

Area requiring further clarification: The authors should provide a clear threshold or criterion for determining which particles are considered high-likelihood to ensure that the ratio of high-likelihood particles is a meaningful measure of optimization progress.

Optimization Strength Control:

The authors propose controlling the optimization strength by combining the ratio of high-likelihood particles pr(m) with the maximum number of iterations. They suggest stopping the optimization process when the ratio of high-likelihood particles in the nth iteration exceeds a set threshold prmax or when the number of iterations exceeds the maximum number of iterations.

Areas requiring further clarification:

1. Selection of prmax: The authors should discuss the factors that influence the choice of the maximum ratio of high-likelihood particles and provide recommendations based on their experience or empirical results.

2. Trade-off between prmax and maximum number of iterations: The authors should provide insight into how these two criteria interact and affect the optimization process, and how to balance them for effective optimization strength control.

3. Premature convergence: The authors should discuss strategies to mitigate the risk of premature convergence, such as incorporating a minimum number of iterations or a diversity maintenance mechanism.

Your review of section 3.1.3 "Particle Density Detection" is thorough and well-organized. You have identified several inconsistencies and areas that require further clarification, which will help improve the scientific rigor and practicality of the proposed approach. Let's summarize the key points and provide a comprehensive review of the section.

REVIEW OF SECTION 3.1.3:

The authors introduce a particle density detection mechanism to measure the particle distribution after iteration and adjust the particle distribution around the optimal particle if the density exceeds a specified threshold. They propose constructing a spring system between the particles in the judgment area and the optimal particle, with the compression of the spring equal to the distance the particle moves towards the optimal particle.

Rebound Coefficient:

Equation (31) defines the rebound coefficient cⁿᵢ as the ratio of the elastic force Fⁿᵢ to an unspecified parameter β. The equation is consistent with the concept of a rebound coefficient, which determines the magnitude of the particle's rebound when the density is too high.

Inconsistencies and areas requiring further clarification:

1. Definition of β: The authors should clarify the meaning of β and its role in the rebound coefficient calculation.

2. Relationship between Equations (31) and (32): The authors should explain how the alternative expression for the rebound coefficient cⁿᵢ in Equation (32) is derived from Equation (31) and how it relates to the previous definition.

3. Selection of constant c: The authors should provide guidance on how to select an appropriate value for the constant c and discuss its impact on the particle distribution adjustment process.

Update of Particle Position under the Action of an Elastic Mechanism:

Equation (33) describes the process of updating particle positions when the particle density exceeds the specified threshold. The particles in the judgment area should rebound in the opposite direction of compression, with the rebound distance determined by the rebound coefficient.

Areas requiring further clarification:

1. Threshold value for particle density: The authors should provide a clear criterion for determining when the particle density is considered too high and requires adjustment.

2. Impact on convergence properties: The authors should provide insight into how the particle position adjustment affects the overall optimization process and whether it may lead to instability or oscillations in certain cases.

3. Computational complexity: The authors should discuss the scalability of the proposed approach, especially for high-dimensional optimization problems or large particle populations.

SECTION 3.2: PARTICLE WEIGHT FILTER COMPENSATION CONTROL DESIGN

The authors introduce the concept of necessary sampling and propose a weight compensation function to ensure the uniformity of the weighted particle distribution before and after optimization. The equations and explanations are generally clear and consistent with the principles of particle filtering and importance sampling.

However, there are a few points that require further clarification:

1. Integration of the exponentially convergent disturbance observer: The authors should provide a clear explanation of how this observer is integrated into the overall control scheme and how it interacts with the particle weight filter compensation.

2. Equation (44): The authors should discuss how the gains kp, ki, and kd are tuned or optimized using the elastic FA algorithm and provide more details on the integration of the FA algorithm with the PID controller tuning process.

Your review of section 3.2 "Particle weight filter compensation control design" is comprehensive and well-structured. You have identified several inconsistencies and areas that require further clarification, which will help improve the scientific rigor and practicality of the proposed approach. Let's summarize the key points and provide a comprehensive review of the section.

Importance Density Function:

The authors define the importance density function g[x(t)] as the probability density function from which the optimized sample set {xⁿᵢ(t)} is drawn, as opposed to the desired posterior probability density function p[x(t)|Z(t-1)]. This concept is consistent with the principles of importance sampling and provides a basis for the weight compensation process.

Weight Compensation Function:

Equation (35) proposes a weight compensation function to ensure the uniformity of the weighted particle distribution before and after optimization. The function is based on the ratio of the posterior probability density function to the importance density function, as shown in Equation (34).

Inconsistencies and areas requiring further clarification:

1. Relationship between R(t)ⁱ and ωⁱ(t): The authors should explain the relationship between the ratio of the posterior probability density function to the importance density function, denoted as R(t)ⁱ in Equation (34), and the weight compensation function, denoted as ωⁱ(t) in Equation (35).

2. Estimation of density functions: The authors should discuss the computational aspects of evaluating the posterior probability density function p[x(t)|Z(t-1)] and the importance density function g[x(t)] in practice, as well as the potential challenges or limitations involved.

3. Integration with particle filter algorithm: The authors should provide a more detailed description of the complete particle filter algorithm with weight compensation and clarify the sequence of steps involved, including how the weight compensation function interacts with the resampling step.

Exponentially Convergent Disturbance Observer:

Equation (36) introduces an exponentially convergent disturbance observer to estimate the system disturbance and compensate for it in the PID controller. The observer gain K is chosen to ensure exponential convergence of the estimation error.

Areas requiring further clarification:

1. Selection of observer gain K: The authors should provide guidance on how to select an appropriate value for the observer gain K and discuss the trade-off between convergence speed and noise sensitivity.

2. Robustness to uncertainties and noise: The authors should analyze the sensitivity of the disturbance observer to model uncertainties and measurement noise, and propose strategies to mitigate their impact on the estimation accuracy.

3. Integration with PID controller: In Figure 4, the authors should clarify how the disturbance estimate from the observer is integrated into the PID controller and how it affects the control signal.

Comments in Figure 4:

The block diagram of the elastic FA algorithm optimized filter adaptive PID control structure in Figure 4 shows the disturbance observer as a separate block without any connections to the other components of the control system. The authors should revise the figure to clearly illustrate how the disturbance estimate is integrated into the PID controller and how it interacts with the other elements of the control structure.

SECTION 3.3: OPTIMIZING PID PARAMETERS USING THE ELASTIC FA ALGORITHM

The authors provide a step-by-step procedure for applying the elastic FA algorithm to tune the PID gains. The optimization steps are clearly explained and consistent with the principles of the elastic FA algorithm introduced earlier.

HOWEVER, there are a few areas that could benefit from further discussion:

1. Computational complexity and convergence properties: The authors should discuss the algorithm's efficiency and scalability, especially for real-time control applications.

2. Comparison with other optimization algorithms and adaptive control techniques: Including such a comparison would help highlight the advantages and limitations of the elastic FA algorithm optimization filter for PID controller tuning.

The authors propose using the elastic firefly algorithm to optimize the PID controller parameters (kp, ki, and kd). They provide a step-by-step procedure for applying the algorithm to tune the controller gains.

Step 1: Initialization

The authors specify the value ranges for the PID parameters based on engineering experience and set the initial values for the algorithm parameters, such as population size, fluorescence brightness, and attraction.

Areas requiring further clarification:

1. Justification for parameter ranges and initial values: The authors should discuss the rationale behind the chosen parameter ranges and initial values, and consider conducting sensitivity analyses to assess their impact on the optimization results.

Step 2: Iteration

The authors describe the iterative process of updating the particle positions based on the attraction and density calculations introduced in the previous sections, which is consistent with the general structure of the firefly algorithm.

Areas requiring further clarification:

1. Termination criteria: The authors should provide clear guidelines on when to stop the optimization process, such as specifying the maximum number of iterations or the convergence threshold.

Step 3: Termination

The optimization process terminates when either the maximum number of iterations is reached or the ratio of high-likelihood particles exceeds a predefined threshold.

Areas requiring further clarification:

1. Trade-off between termination criteria: The authors should analyze the impact of the maximum number of iterations and the high-likelihood particle ratio threshold on the optimization results and propose strategies for balancing the exploration and exploitation aspects of the algorithm.

Optimization Results:

The elastic firefly algorithm reached the termination condition after 67 iterations, resulting in the optimized PID parameters: kp = 47.832, ki = 0.718, and kd = 0.697.

Areas requiring further analysis and discussion:

1. Comparison with other methods: The authors should benchmark their approach against existing methods to highlight its advantages and limitations.

2. Robustness to variations: The authors should analyze the sensitivity of the controller's performance to parameter uncertainties and propose strategies for ensuring robust and stable operation.

3. Computational complexity and convergence properties: The authors should discuss the scalability of their approach and its suitability for real-time implementation on resource-constrained platforms.

REVIEW OF SECTION 4.1:

The authors present the establishment of a simulation model for the mining concrete mixing and transport tank truck using a state-space representation and a schematic diagram. They also provide a detailed list of system parameters that characterize the vehicle's physical properties.

State-Space Model:

Equation (47) introduces a state-space model of the system, where the state variable x includes the vehicle speed ξ, acceleration dξ/dt, and other related variables. The model is expressed as a matrix differential equation, with coefficient matrices M, C, and N, and an additional term w representing the relative motion of each order of mass in the equivalent system.

Areas requiring further clarification:

1. Derivation of coefficient matrices: The authors should discuss the process of system identification and the assumptions made in obtaining the coefficient matrices M, C, and N from the physical properties of the mining concrete mixing and transport tank truck.

Simulation Model Schematic:

Figure 5 presents a schematic diagram of the dynamic simulation model, including an equivalent mechanical model of liquid sloshing, a dynamic model of the concrete mixing transport tanker, and a display of the vehicle's lateral acceleration.

Areas requiring further clarification:

1. Definition of variables: The authors should provide a clear definition of the variables U, θ, and ω in the schematic and explain their role in the simulation model.

2. Integration of liquid sloshing and vehicle dynamics: The authors should clarify how the equivalent mechanical model of liquid sloshing and the dynamic model of the concrete mixing transport tanker are integrated and how the liquid sloshing dynamics influence the overall vehicle dynamics.

3. Computational tools and considerations: The authors should provide information on the software or programming environment used to implement the simulation model and discuss any specific considerations for numerical integration or solver settings.

System Parameters:

Table 1 lists the system parameters of the mining concrete mixing and transport tank truck, including its dimensions, weight, engine specifications, and other relevant properties.

Areas requiring further discussion:

1. Source and methodology: The authors should clarify the source or methodology used to obtain the listed parameters, whether they are based on manufacturer specifications, experimental measurements, or estimates from similar vehicles.

2. Sensitivity analysis: The authors should analyze the sensitivity of the model's predictions to changes in key parameters and propose strategies for robust simulation and design.

3. Validation and verification: The authors should compare the simulated vehicle's performance with experimental measurements or field observations to assess the accuracy and reliability of the simulation model.

REVIEW OF SECTION 4.2:

The authors present a control simulation system experiment for the mining concrete mixing tank truck using MATLAB/Simulink. They develop a multi-factor simulation model that integrates the tank truck dynamics, hydrostatic drive system, rock slope model, and the elastic FA algorithm optimized filter adaptive PID controller. The authors conduct simulation experiments to compare the performance of the proposed controller with the traditional PID controller under various operating conditions.

Simulation Model Development:

The development of a comprehensive simulation model that considers multiple factors affecting the mining concrete mixing tank truck's performance is a significant contribution of this study. The model incorporates the vehicle dynamics, hydrostatic drive system, rock slope model, and the proposed adaptive PID controller optimized by the elastic FA algorithm.

Areas requiring further clarification:

1. Detailed description of model architecture: The authors should provide a more detailed description of the model architecture and the key Simulink blocks employed to enhance the reproducibility of their work.

Step Response Experiment (Figure 7):

The results demonstrate that the proposed controller achieves a faster settling time (5.4s) and a lower steady-state error (0.01km/h) compared to the traditional PID controller, while also eliminating overshoot.

Areas requiring further discussion:

1. Selection of step signal amplitude: The authors should provide more information on the selection of the step signal amplitude and the rationale behind it.

2. Implications of improved performance: The authors should discuss the implications of the improved settling time and steady-state error on the overall performance and efficiency of the mining concrete mixing tank truck.

Ramp Driving Experiment (Figure 8):

The results show that the proposed controller maintains a smaller speed tracking error (0.04 km/h) compared to the traditional PID controller (0.22 km/h) when entering and exiting the ramp, demonstrating faster convergence and better disturbance rejection capabilities.

Areas requiring further clarification:

1. Ramp profile and duration: The authors should provide more details on the ramp profile and the duration of the ramp driving experiment.

2. Impact on safety and productivity: The authors should discuss the impact of the improved speed tracking performance on the safety and productivity of the mining operation, considering the challenges posed by steep slopes and uneven terrain.

Comprehensive Driving Experiment (Figure 9):

The results demonstrate that the proposed controller maintains a lower average speed control error (0.03 km/h) and maximum speed error (0.17 km/h) compared to the traditional PID controller, while also exhibiting better disturbance rejection and adaptability to varying road conditions.

Areas requiring further discussion:

1. Characteristics of white noise interference: The authors should provide more information on the characteristics of the white noise interference and its relevance to real-world driving conditions.

2. Implications of improved performance: The authors should discuss the implications of the improved speed control accuracy and robustness on the overall efficiency and reliability of the mining concrete mixing tank truck.

Dynamic Disturbance Experiment (Figure 10):

The results show that the proposed controller maintains a smaller speed tracking error (within 0.3 km/h) compared to the traditional PID controller (up to 0.47 km/h) when subjected to fluid sloshing disturbances, demonstrating better adaptability and disturbance rejection capabilities.

Areas requiring further clarification:

1. Modeling of fluid sloshing disturbance: The authors should provide more details on the characteristics of the fluid sloshing disturbance and how it is modeled in the simulation.

2. Impact on stability and safety: The authors should discuss the potential impact of fluid sloshing on the stability and safety of the mining concrete mixing tank truck, and highlight the significance of the improved speed tracking performance in mitigating these risks.

Criticism and Suggestions:

1. Detailed information on simulation model implementation: The authors should provide more detailed information on the implementation of the simulation model in MATLAB/Simulink, including the specific blocks and libraries used for each component, to enhance the reproducibility and transparency of their work.

2. Discussion of simulation parameters: The authors should discuss the selection of the simulation parameters, such as the step signal amplitude, ramp profile, and white noise characteristics, and justify their relevance to real-world operating conditions.

3. Comprehensive analysis of implications: The authors should provide a more comprehensive analysis of the implications of the improved control performance on the safety, productivity, and efficiency of the mining operation, relating the simulation results to the specific challenges and requirements of mining concrete mixing tank trucks.

4. Sensitivity analyses: The authors should consider conducting sensitivity analyses to evaluate the robustness of the proposed controller to variations in system parameters, such as vehicle mass, tire properties, and fluid characteristics, to provide insights into the controller's adaptability to different operating scenarios.

5. Comparison with state-of-the-art control techniques: The authors should compare the performance of the proposed controller with other state-of-the-art control techniques for mining vehicles, such as model predictive control or sliding mode control, to benchmark the effectiveness of the elastic FA optimized filter adaptive PID controller against existing approaches.

REVIEW OF SECTION 4.3:

The authors present the verification of the simulation model for the KJCJ-4 mining concrete mixing and transport tank truck by comparing the simulation results with actual vehicle test data under a single lane shifting condition. They assess the accuracy and reliability of the established simulation model by examining the lateral acceleration curves obtained from the simulation and the actual vehicle test.

Verification Scenario:

The authors consider a single lane shifting condition with a filling rate of 1.5 and a vehicle speed of 50 km/h to verify the simulation model.

Areas requiring further clarification:

1. Justification for chosen scenario: The authors should provide more justification for choosing the single lane shifting condition and the specific filling rate and vehicle speed, discussing their relevance to the typical operating range of the mining concrete mixing and transport tank truck.

Lateral Acceleration Comparison (Figures 11(a) and 11(b)):

The results show that the simulation curve and the test curve exhibit good consistency in both the change trend and the range of lateral acceleration values, with a relative error within 7.2%.

Areas requiring further analysis:

1. Quantitative metrics for agreement: The authors should provide more quantitative metrics, such as the root mean square error (RMSE) or the coefficient of determination (R^2), to assess the agreement between the simulation and the test data objectively.

Fluid Sloshing Interference (Figure 11(b)):

The results demonstrate that the simulation model accurately reproduces the lateral acceleration profile under fluid sloshing interference, with good agreement in both the change trend and the range of values.

Areas requiring further clarification:

1. Modeling of fluid sloshing interference: The authors should provide more details on how the fluid sloshing interference is modeled in the simulation and how it is introduced in the actual vehicle test.

2. Impact on stability and safety: The authors should discuss the potential impact of fluid sloshing on the stability and safety of the mining concrete mixing tank truck and highlight the importance of accurately capturing these effects in the simulation model.

Criticism and Suggestions:

1. Justification for verification scenario selection: The authors should provide more justification for the selection of the single lane shifting condition and the specific filling rate and vehicle speed used for verification, discussing their representativeness and relevance to the typical operating range of the mining concrete mixing and transport tank truck.

2. Quantitative metrics for agreement: The authors should include more quantitative metrics, such as the root mean square error (RMSE) or the coefficient of determination (R^2), to assess the agreement between the simulation and the test data objectively and provide a standardized measure of the simulation model's accuracy.

3. Verification under diverse operating conditions: The authors should consider verifying the simulation model under a wider range of operating conditions, such as different filling rates, vehicle speeds, and road profiles, to demonstrate the robustness and versatility of the simulation model in capturing the vehicle dynamics under diverse scenarios.

4. Experimental setup and data acquisition details: The authors should provide more details on the experimental setup and the data acquisition system used for the actual vehicle test, discussing the sensors used, the sampling rate, and any signal processing techniques applied to ensure the reliability and consistency of the test data.

5. Analysis of discrepancy sources: The authors should analyze the sources of discrepancy between the simulation and the test results, such as modeling assumptions, parameter uncertainties, and measurement errors, discussing their potential impact on the simulation model's accuracy and proposing strategies for minimizing their effects.

Your critical review of the "Conclusion and Outlook" section provides a comprehensive analysis of the main contributions, findings, and potential improvements in the paper. You have summarized the key points discussed by the authors, identified missing elements in the outlook, and highlighted inconsistencies with the main idea of the paper. Let's organize your review into a structured format to enhance clarity and readability.

REVIEW OF SECTION 5:

Missing Elements in the Outlook:

The authors do not explicitly provide an outlook or future research directions. They could have discussed potential extensions of their work, such as:

1. Experimental validation of the proposed control algorithm on a real concrete mixing tank truck.

2. Integration of the control algorithm with advanced sensing and monitoring technologies.

3. Investigation of the long-term durability and reliability of the hydrostatic drive system components under the proposed control scheme.

4. Extension of the research to other types of heavy-duty vehicles used in open-pit mines.

Inconsistencies and Problems:

1. Lack of a clear and concise summary of the main objectives and methodologies.

2. Insufficient explanation of how the ground mechanics and hydrostatic drive system models support the development and evaluation of the proposed control algorithm.

3. Missing explicit link between the liquid sloshing dynamics findings and the adaptive control algorithm's performance.

4. Absence of a clear statement of the practical implications and potential benefits of the proposed algorithm for open-pit mining operations.

Recommendations:

1. Include a brief restatement of the research problem and the proposed solution approach at the beginning of the conclusion section to help readers recall the main idea of the paper.

2. Explain how the ground mechanics and hydrostatic drive system models support the development and evaluation of the proposed control algorithm.

3. Highlight how the proposed algorithm helps mitigate the adverse effects of liquid sloshing on vehicle stability and safety.

4. Emphasize the practical implications and potential benefits of the proposed adaptive hydraulic drive control algorithm for open-pit mining operations.

5. Provide a clear outlook on future research directions, addressing the identified missing elements.

By incorporating these recommendations, the authors can enhance the clarity, coherence, and impact of the "Conclusion and Outlook" section, ensuring that it effectively summarizes the main contributions and findings of the research while maintaining consistency with the main idea of the paper.
