# Supplementary material for: Research on adaptive hydraulic drive optimization control of concrete mixing tank truck for open-pit mine
Source: PLoS One. 2024 Oct 9;19(10):e0310249. doi: 10.1371/journal.pone.0310249 (PMC11463772; doi:10.1371/journal.pone.0310249)
Supplement: Attachment — Submitted filename: Response to Reviewers 2.docx [file pone.0310249.s004.docx]

Dear Editor and Reviewer:

Thank you for your letter and your reviewer's comments on our paper "Study on Adaptive Hydraulic Drive Optimization Control of Open-Pit Mine Concrete Mixer Tank Truck" (PONE-D-24-25486). These comments are very valuable and very helpful for us to revise and improve the paper, and also have important guiding significance for our research. We carefully studied the comments and made revisions, hoping to get approval. At the same time, we are very grateful to PLOS ONE for providing a good communication platform for researchers! I wish the journal will continue to do better!

Dear Authors here is the comments for the manuscript as follows:

ABSTRACT:

Your review of the abstract provides a thorough analysis of its content, structure, and effectiveness in conveying the key aspects of the research. Let's organize the review into a more structured format to enhance clarity and readability.

Title: Abstract Review

Language and Consistency:

The abstract is well-written and free of any major language errors. The flow of ideas is logical, and the content is consistent with the title and the expected structure of an abstract. However, there are a few minor suggestions for improvement:

1. Clarify the referent of "it" in the sentence "Then, it analyzes its hydraulic drive control characteristics and structural applications" (presumably referring to the proposed method).

2. Rephrase the sentence "Under filling rates of 1.5 and 2.0, two modes of step steering and double lane change are simulated, respectively" to improve clarity, such as "Step steering and double lane change modes are simulated under filling rates of 1.5 and 2.0, respectively."

Recommendations:

1. Clarify the referent of "it" in the sentence discussing the analysis of hydraulic drive control characteristics and structural applications.

Answer 1. "it" refers to the proposed method. I apologize for the language misunderstanding caused by translation problems. Now I will adjust the language here in the article to make it more uncontroversial.

2. Rephrase the sentence about the simulation of step steering and double lane change modes under different filling rates to improve clarity.

Answer 2.Thank you for your suggestion to rephrase to improve clarity. We believe this will better highlight the topic and give readers a clearer reading experience. Therefore, we adopt the sentence you recommended to us: "Step steering and double lane change modes are simulated under filling rates of 1.5 and 2.0, respectively."

INTRODUCTION:

The authors highlight the challenging environmental conditions in open-pit mines and the safety issues faced by mining concrete mixing tank trucks, particularly the non-axisymmetric problems caused by fluid sloshing in the tank and its impact on vehicle dynamics when coupled with excitations from complex road surfaces.

there are several areas where the introduction could be improved:

1. Insufficient background information: While the authors describe the general conditions in open-pit mines, they do not provide a clear connection between these conditions and the specific problem of fluid sloshing in concrete mixing tank trucks. They should explicitly state how these conditions contribute to the fluid sloshing problem or make it more challenging to address.

Answer 1. In the second paragraph of "INTRODUCTION", a statement is added to clarify the relationship between the environment, road conditions, climate and the specific problem of liquid sloshing in concrete mixer trucks. It is clearly stated that the most important problem is the road condition, and a detailed description is given of how these conditions lead to liquid sloshing or make it more difficult to solve the problem. This can better follow up the following sentence "For such problems, they can be solved by structural improvement or control method optimization. At present, there are adjustable advanced suspension systems [8,9], intelligent transmission systems [10,11], and remote diagnosis systems [12,13] that can adapt to changing terrain conditions and have excellent performance in passing obstacles and slopes..."

2. Limited discussion of existing research: The introduction would benefit from a more comprehensive review of existing literature on the topic. The authors briefly mention potential solutions, such as advanced suspension systems, intelligent transmission systems, and remote diagnosis systems, but do not provide specific examples or references to support their claims. They also state that these solutions are insufficient without a clear explanation of why they are inadequate. A more detailed discussion of existing solutions and their limitations, supported by relevant references, would help readers assess the novelty and significance of the proposed research.

Answer 2. After the major revision, I consulted more references related to this article, and was able to better explain and introduce the current research status at home and abroad. For advanced suspension systems, intelligent transmission systems, and remote diagnosis systems, we also provided some domestic and foreign references to support our views, which would be more convincing. Most importantly, we also supplemented the limitations of these traditional solutions on this basis, and used relevant references to support them, and then took over the optimized adaptive hydraulic drive system proposed below, which would be clearer and easier for readers to read.

3. Lack of clarity in research objectives: The authors state that their main objective is to analyze the stress conditions of mining concrete mixing tank trucks and optimize the control performance to improve driving stability, high-speed accuracy, and robustness. However, this statement is not clearly connected to the identified problem of fluid sloshing and its impact on vehicle dynamics. The authors should revise this statement to clearly articulate how their research objectives address the limitations of existing solutions and the specific problems they have identified.

Answer 3. In Chapter 3 of this article, the previous statement is that the traditional PID control algorithm will make it difficult to meet the requirements of driving stability, high speed accuracy and strong robustness of mining concrete mixing and transport tank trucks under complex working conditions, and then the FA optimized particle filter adaptive PID controller based on elastic mechanism is proposed. There is no clear connection between them. On this basis, we supplemented how the traditional algorithm shows limitations in the face of fluid sloshing problems, and then proposed the improved algorithm of this article below. This can more clearly explain how the research goal of this article solves the limitations of traditional methods, so that readers can understand this article more thoroughly.

4. Inconsistency in problem description: There is an inconsistency between the abstract and the introduction regarding the cause of the non-axisymmetric problem. In the abstract, the authors mention that the non-axisymmetric problem is caused by fluid sloshing in the tank during the driving process, while in the introduction, they state that the fluid sloshing is affected by the excitation of the complex road surface, which makes the fluid sloshing coupled with the body dynamics. This inconsistency should be clarified to ensure a clear understanding of the problem.

Answer 4. We are very sorry for the inconsistency between the abstract and the introduction regarding the causes of the non-axisymmetric problem. We have corrected the sentence error in the abstract and changed it to "the non-axisymmetric problem caused by the sloshing of the fluid in the tank of the mining concrete mixing transport tank truck during driving is affected by the excitation of the complex road surface on the vehicle." In this way, the causes of the non-axisymmetric problem in the abstract and the introduction are consistent. After clarifying the dispute here, we can ensure a clear understanding of the problem.

5. Insufficient justification for the proposed method: The authors introduce their proposed method, the firefly filter compensation optimization adaptive control algorithm with an elastic mechanism, without providing sufficient background information or justification. They should include a brief explanation of these concepts, their advantages over other potential approaches, and why they are suitable for addressing the hydraulic drive control problem in mining concrete mixing tank trucks. Additionally, the references cited in this section are not properly introduced or discussed, making it difficult for readers to understand their relevance to the current study.

Answer 5. For the firefly filter compensation optimization adaptive control algorithm based on elastic mechanism proposed in this paper, we added more background information and arguments to explain these concepts, and explained their advantages over traditional methods and the reasons why they are applicable in this engineering context. Finally, we improved the references and added many international references to make the article more comprehensive and complete.

6. Lack of research questions or hypotheses: The introduction does not explicitly state the research questions or hypotheses that the study aims to address. Clearly defining these would help readers understand the specific goals and expectations of the research.

Answer 6. In the "INTRODUCTION" section, we have improved the problems and assumptions of concrete mixing and transport tank trucks used in open-pit mines at this stage, which makes the research of this article more necessary and helps readers to have a deeper understanding of this article.

A deeper analysis of the introduction by quoting specific passages and providing a critical assessment.

Quote 1: "The mining site of an open-pit mine is usually vast, with an open working surface and complex environmental conditions in the mining area. It often faces harsh climatic conditions, such as high temperatures, cold, wind, and sand. In addition, the working range of an open-pit mine is extensive, the working surface often changes, the road conditions are relatively complex, the transportation distance is long, and the transportation task is heavy [1-6]."

comment: This passage sets the context for the research problem by describing the challenging conditions in open-pit mines. However, the authors do not provide a clear connection between these conditions and the specific problem of fluid sloshing in concrete mixing tank trucks. They could improve this by explicitly stating how these conditions contribute to the fluid sloshing problem or make it more challenging to address.

Answer 1. We added the most prominent conditions that cause fluid sloshing in concrete mixer trucks in the second paragraph of the "INTRODUCTION" section of this article, which can better enhance the connection between them. This can better follow the following text "For such problems, they can be solved by structural improvement or control method optimization. At present, there are adjustable advanced suspension systems [8,9], intelligent transmission systems [10,11], and remote diagnosis systems [12,13] that can adapt to changing terrain conditions and have excellent performance in passing obstacles and slopes..."

Quote 2: "For such problems, they can be solved by structural improvement or control method optimization. There are adjustable advanced suspension systems, intelligent transmission systems, and remote diagnosis systems that can adapt to changing terrain conditions and perform excellently in passing obstacles and slopes. However, the above solutions are still insufficient to solve the driving problems of mining concrete mixing tank trucks in complex environments such as uphill and sloped roads."

comment: The authors mention potential solutions, such as structural improvements and control method optimization, but they do not provide specific examples or references to support their claims. They also state that existing solutions are insufficient without providing a clear explanation of why they are inadequate. This lack of detail makes it difficult for readers to assess the novelty and significance of the proposed research.

Answer 2. We have consulted more references related to this article, and can better explain and introduce the current research status at home and abroad. For these existing systems, we also provide some references at home and abroad to support our views, which will be more convincing. Then, on this basis, we also supplemented the limitations of these traditional solutions and used relevant references to support them, and then took over the optimized adaptive hydraulic drive system proposed below.

Quote 3: "Therefore, analyzing the stress conditions of mining concrete mixing tank trucks and optimizing the control performance effect [8-10]."

Comment: This sentence is incomplete and lacks clarity. The authors should revise it to clearly state the objective of their research and how it addresses the limitations of existing solutions. Additionally, the references [8-10] are not properly introduced or discussed, making it difficult for readers to understand their relevance to the current study.

Answer 3. We supplemented this sentence with the following: Through in-depth analysis of the force applied to vehicles under complex road conditions, we aim to improve the adaptive hydraulic drive system to solve the problem that traditional solutions are prone to overturning and other accidents under complex working conditions such as uneven roads and slopes. The following is an explanation and introduction to the current status of research at home and abroad.

Quote 4: "In order to improve the hydraulic drive control of a concrete mixer transport tank truck for mining, this paper adopts the firefly filter compensation optimization adaptive control algorithm [16-19] and introduces an elastic mechanism [20] to speed up the optimization process."

Comment: The authors introduce their proposed method without providing sufficient background information on the firefly filter compensation optimization adaptive control algorithm and the elastic mechanism. They should include a brief explanation of these concepts and why they are suitable for addressing the hydraulic drive control problem in mining concrete mixing tank trucks. Furthermore, the references [16-20] are not properly discussed, and their relevance to the current study is not clearly established.

Answer 4. We have added more background information and arguments to explain these concepts in the firefly filter compensation optimization adaptive control algorithm based on elastic mechanism proposed in this paper, and explained their advantages over traditional methods and the reasons why they are applicable to mining concrete mixing tank trucks. Finally, we have improved the references and added many international references.

The authors should address the identified inconsistencies, provide a clearer problem statement, and better justify their proposed approach. They should also include a more comprehensive review of existing literature, clearly define their research objectives and hypotheses, and establish a stronger connection between the challenging conditions in open-pit mines and the specific problem of fluid sloshing in concrete mixing tank trucks. By addressing these issues, the authors can strengthen the introduction and provide a more solid foundation for the rest of the paper.

REVIEW OF SECTION 2.1:

The authors aim to study the motion state of a mining concrete mixing and transport tank truck driving on a sloped platform by conducting a force analysis of the vehicle body.

Quote 1: "In order to study the motion state of a mining concrete mixing and transport tank truck when it is driving on a platform with a slope angle, the force analysis of the vehicle body is carried out, as shown in Fig. 1, where L₁,L₂ the distances along the slope direction between the center of mass of the concrete tank truck and the contact points between the front and rear tires and the road slope, respectively, and H is the average distance between the center of mass and the slope."

Comment: The authors clearly describe the purpose of this section and introduce the variables L₁, L₂, and H, which are used in the force analysis of the vehicle body on a sloped platform. The explanation is concise and easy to understand. Therefore, no need to fix anything.

Answer 1: Thank you for your recognition of this section of the article. We also hope that other chapters can also receive such affirmation and recognition from you. We will continue to work hard to write this article and every subsequent article.

Quote 2: "According to the static equilibrium equation, we can get: Ft1 + Ft2 + Ft3 = mgsinθ (1) Fn1 + Fn2 + Fn3 = mgcosθ (2)"

Comment: Equations (1) and (2) represent the static equilibrium equations for the forces acting on the vehicle body in the tangential and normal directions. The equations are consistent with the force diagram shown in Fig. 1. HOWEVER, the authors introduce Ft3 and Fn3 without prior definition, which may confuse readers. It would be better to clarify that these forces represent the tangential and normal forces acting on the third axle of the vehicle.

Answer 2: We are sorry that due to our negligence in formula editing, we introduced two parameters $F_{\text{t3}}$ and $F_{\text{n}3}$ that were not defined in advance. After unanimous discussion among our authors, we deleted the two redundant parameters and improved the formula.

$$F_{\text{t}1}+F_{\text{t}2}=mg\sin\theta$$

$$F_{\text{nl}}+F_{\text{n}2}=mg\cos\theta$$

Where: $F_{\text{t}i}$ is the vehicle traction/N; $F_{\text{n}i}$ is the vehicle positive pressure/N; $m$ is the mass of the transport tanker/kg; $g$ is the acceleration of gravity/(m/s²); $\theta$ is the slope angle.

Quote 3: "The mining concrete mixing transport tanker is driving on the slope platform, and the interaction between the wheels and the slope is shown in Fig. 2."

Comment: The authors introduce a new force interaction model between the wheels and the slope platform, which is essential for understanding the vehicle's dynamics on the slope. The description is clear and supported by a visual representation in Fig. 2.

Answer 3: This sentence and Figure 2 below introduce a new dynamic model of a concrete mixing tank truck for mining between the wheels and the slope platform. It can not only vividly and intuitively show the force of the KJCJ-4 concrete mixing tank truck for mining on the slope platform of the open-pit mine, but also pave the way for the subsequent study of the hydraulic drive system model, providing readers with a reading experience of combining pictures and texts. Thank you for your recognition of this section, and we also hope that other chapters can also receive your affirmation and recognition.

Equations (3) to (8):

Comment: Equations (3) to (8) describe various force interactions and parameters related to the wheel-slope interaction, such as the approach angle, maximum stress point approach angle, normal stress, and shear stress. The equations are derived from well-established models, such as the Bekker model for terrain-vehicle interaction. The authors have provided clear explanations for each equation and the variables involved.

HOWEVER, THERE ARE A FEW INCONSISTENCIES AND AREAS THAT REQUIRE FURTHER CLARIFICATION:

1. In Equation (6), the shear stress τ is expressed as a function of the normal stress σ, but σ is not included in the equation. The authors should clarify this inconsistency and ensure that all variables are properly defined.

Answer 1. We have detailedly marked and defined the normal stress $\sigma$ and other parameters in formula (6) so that readers can better understand their meanings.

2. In Equation (7), the authors use θ₁ and θₘ without prior definition. They should clearly explain the meaning of these variables to avoid confusion and improve the equation's readability.

Answer 2. We have detailedly labeled and defined the parameters $\alpha_{1}$ and $\alpha_{\text{m}}$ in the newly added formula (7) and the parameters in formulas (8) and (9), so that readers can better understand their meanings.

$$j(\theta)=r[\alpha_{1}-\alpha_{\text{m}}-(1-s)(\sin\alpha_{1}-\sin\alpha_{\text{m}})]$$

$$F_{\text{n}}=\frac{rw[\sigma(\alpha_{1}\cos\alpha_{\text{m}}-\alpha_{\text{m}}\cos\alpha_{1}-\alpha_{1}+\alpha_{\text{m}})+ \tau(\alpha_{1}\sin\alpha_{\text{m}}-\alpha_{\text{m}}\sin\alpha_{1})]}{\alpha_{\text{m}}\left(\alpha_{1}-\alpha_{m}\right)}$$

$$F_{\text{t}}=\frac{rw[\tau(\alpha_{1}\cos\alpha_{\text{m}}-\alpha_{\text{m}}\cos\alpha_{1}-\alpha_{1}+\alpha_{\text{m}})+ \sigma(\alpha_{1}\sin\alpha_{\text{m}}-\alpha_{\text{m}}\sin\alpha_{1})]}{\alpha_{\text{m}}\left(\alpha_{1}-\alpha_{m}\right)}$$

Among them: is the inherent shear characteristic parameter of rock, which represents cohesion/N and internal friction angle respectively; $\sigma$ is normal stress/N; $j(\theta)$ is the shear displacement of soil/m; $\alpha_{1}$ is the entry angle of wheel thorn effect; $\alpha_{\text{m}}$ is the wheel-ground action angle; $s$ is the slip rate.

3. The authors introduce the tire sinkage model in Equation (9) but do not provide a clear explanation of how this model relates to the overall mechanical model of the slope platform. They should clarify the significance of the tire sinkage model and its role in the analysis.

Answer 3. The meaning and application scenarios of the wheel static subsidence model are supplemented above formula (9), and its application fields are explained, "The wheel static subsidence model is used to calculate the contact pressure distribution between the wheels of the mining concrete mixing and transport tank and the ground...", and the relationship between the model and the overall mechanical model of the slope platform is further explained, making this paragraph more closely related and enabling readers to have a clearer understanding of the article.

4. The tire sinkage model in Equation (9) includes several parameters, such as b, kc, and kφ, which are not defined in the text. The authors should provide definitions and explanations for these parameters to ensure that readers can fully understand the equation.

Answer 4. During the Major Revision, we supplemented and improved the definitions and meanings of several parameters such as, $b$, $k_{\text{c}}$, and in formula (10) so that readers can better understand their meanings.

$$Z_{i}=\left[\frac{3Y}{bk\sqrt{2r}\left(3-n\right)}\right]^{\frac{2}{2n+1}}$$

Among them: $Y$ is the wheel load; $k$ is the coefficient; $b$ represents the parameter related to the geometry, structure or material properties of the tire; $k_{\text{c}}$ represents the coefficient related to the stiffness, elasticity or load-bearing capacity of the tire; represents the variable related to the tire characteristics or usage conditions.

5. In Equation (10), the authors introduce the sinking coefficient λ to represent the relationship between the front and rear wheel sinkage. However, they do not provide a clear explanation of how this coefficient is determined or its physical significance in the context of the mining concrete mixing transport tanker. Additional information on the sinking coefficient would improve the reader's understanding of the mechanical model.

Answer 5. In the context of formula (11), we have supplemented and improved the brief physical meaning of the subsidence coefficient. However, due to the length of the article and the need to keep the content concise, we use the reference citation method to explain more determination methods and descriptions and details of the subsidence coefficient, so that readers can have a deeper understanding of the mechanical model.

REVIEW OF SECTION 2.2:

The authors describe the closed hydraulic drive system used in most mining concrete mixing and transport tank trucks, supported by a schematic representation in Fig. 3. The explanation of the system's components and their functions is clear and concise, allowing readers to understand the system's overall structure and operation.

Quote 1: "Most mining concrete mixing and transport tank trucks use a closed hydraulic drive system, the structure of which is shown in Fig. 3. The oil ports at both ends of the variable pump are connected end to end with the oil inlet and return ports of the quantitative motor to form a closed loop."

Comment: The description of the closed hydraulic drive system is clear and supported by Fig. 3, which enhances the reader's understanding of the system's layout.

Answer 1: Thank you for your recognition of the closed hydraulic drive system section. This section will be more convincing if the system is described in detail in words and supported by Figure 3. Doing so will help readers better understand the content.

Quote 2: "The engine drives the variable pump to provide high-pressure oil to the system, and the variable mechanism can be adjusted according to the control signal to achieve the speed and steering control of the quantitative motor. The system oil replenishment circuit consists of a one-way valve, an accumulator, an oil replenishment pump, an oil tank, and a flow valve to replenish the leaked oil in time for the system to prevent cavitation."

Comment: The explanation of the system's functioning is concise and informative, covering the roles of key components such as the variable pump, variable mechanism, and oil replenishment circuit.

Equations (13) to (16):

Comment: The equations presented in this section are consistent with the fundamental principles of fluid mechanics, rotational dynamics, and gear ratios.

Answer 2: This sentence mainly explains that the variable displacement pump and the fixed displacement motor form a closed loop, which is driven by the engine and adjusts the speed and direction according to the control signal. The oil replenishment circuit includes a check valve, an accumulator, an oil replenishment pump, an oil tank and a flow valve to prevent cavitation. This allows readers to understand the basic overview of this system and paves the way for the introduction of the elastic mechanism and the firefly algorithm below.

HOWEVER, there are some inconsistencies and areas that require further clarification:

1. Units: The authors do not provide units for the variables used in the equations, which may lead to confusion and difficulty in reproducing the results. They should include the appropriate units for each variable to ensure clarity and reproducibility.

Answer 1. The original formulas (13) to (16) have been supplemented and improved, and are now formulas (15) to (18). We have supplemented and improved all the parameter symbols and units in these formulas, as well as the units of the formulas in other parts of the article. The following is the formula after our improvement:

$$q_{\text{P}}=k_{\text{P}}\omega_{\text{P}}u-C_{\text{ip}}(p_{1}-p_{2})-C_{\text{ep}}p_{1}$$

Among them: $q_{\text{P}}$ is the output flow of the variable pump/(m³/s); $k_{\text{P}}$, $\omega_{\text{P}}$, and $u$ are the displacement gain/(m³/s), speed/(r/s), and control amount of the variable pump respectively; $C_{\text{ip}}$ and $C_{\text{ep}}$ are the internal and external leakage coefficients of the variable pump/(m³/s/Pa); $p_{1}$ and $p_{2}$ are the inlet and outlet pressures of the quantitative motor respectively/Pa.

Among them: $q_{\text{M}}$ is the motor input flow/(m³/s), which is equal to the pump output flow; $C_{\text{im}}$ and $C_{\text{em}}$ are the motor internal and external leakage coefficients/(m³/s/Pa); $D_{\text{m}}$ is the motor displacement/m³; $\omega_{\text{m}}$ is the motor speed/(r/s); $V_{0}$ is the motor oil inlet volume/m³; $\beta_{\text{e}}$ is the effective elastic modulus of the oil/Pa.

Where: $J_{\text{t}}$ is the moment of inertia of the motor and drive axle/(kg·m²); $B_{\text{m}}$ is the rotation damping coefficient; $T_{\text{L}}$ is the load torque/(N·m).

$$\nu=\omega_{\text{m}}R_{\text{t}}i_{\text{v}}$$

Where: $R_{\text{t}}$ is the tire radius/m, $i_{\text{v}}$ is the speed ratio of the total gear.

2. Assumptions and limitations: The authors do not discuss the assumptions or limitations (try to refer to previous works) of the hydraulic drive system model. They should acknowledge any simplifications or approximations made in deriving the equations and discuss their potential impact on the model's accuracy. This would help readers understand the model's applicability and limitations.

Answer 2. By reviewing past research materials, we objectively evaluated the pros and cons of the hydraulic drive system model from various angles. This way of talking will be more realistic and substantial. We also supplemented and improved the derivation process of each formula in the process of explaining the formula. This will make the reader's thinking more coherent and can better refer to the central idea of this article.

3. Load torque: In Equation (15), the load torque TL is not clearly defined or explained. The authors should provide more information on how this torque is determined and what factors influence its value. This would enable readers to better understand the torque balance equation and its implications for the hydraulic drive system.

Answer 3. We apologize that our question did not provide a detailed definition and explanation of the key parameter load moment $T_{\text{L}}$. The original formula (15) was changed to the latest formula (17). We further discussed this parameter, discussed the specific factors that affect the change of this parameter, and the effect of this parameter on the system of this article. Finally, we cited references to make this description more convincing.

4. Gear ratio: In Equation (16), the authors should specify whether the gear ratio iv is the overall gear ratio or the ratio of a specific gear pair in the transmission. Clarifying this would help readers interpret the relationship between motor speed and vehicle speed more accurately.

Answer 4. The original formula (16) has been supplemented and improved to become formula (18). After unanimous discussion among our authors, the parameter $i_{\text{v}}$ is the speed ratio of the total gear, rather than a specific gear ratio. More detailed modifications and definitions have been made here, which will give readers a more detailed understanding of this.

5. Bulk modulus: In Equation (14), the authors use the term "effective bulk modulus" (βe) without providing a clear explanation of what it represents or how it is determined. They should offer a brief description of the bulk modulus and its significance in the flow continuity equation.

Answer 5. The original formula (14) has been supplemented and improved to become formula (16). After unanimous discussion among our authors, we have described and explained the parameter $\beta_{\text{e}}$, the effective elastic modulus of oil, in detail, and explained its significance in the flow continuity equation.

Your critical analysis of section 3 "Design of adaptive PID controller based on elastic FA algorithm optimization filter" is comprehensive and well-structured. You have examined the content, equations, and algorithms, providing insights into the consistency, scientific accuracy, and clarity of the presented material. Let's build upon your analysis and address the inconsistencies and areas that require further clarification.

REVIEW OF SECTION 3:

The authors propose an adaptive PID controller based on the elastic FA algorithm optimization filter to overcome the limitations of traditional PID control in handling the complex driving conditions of mining concrete mixing tank trucks. They provide a clear explanation of the motivation behind their proposed approach and its potential benefits.

SECTION 3.1: OPTIMIZATION PRINCIPLE OF THE ELASTIC FA ALGORITHM

The authors describe the optimization principle of the elastic FA algorithm, including the gravity and movement mechanisms, elasticity optimization strength control, and particle density detection. The explanations of the concepts and equations involved, such as particle fluorescence brightness, particle attraction, and particle position update, are detailed and consistent with the principles of the FA algorithm and the elastic mechanism.

HOWEVER, THERE ARE A FEW INCONSISTENCIES AND AREAS THAT REQUIRE FURTHER CLARIFICATION:

1. Equation (23): The authors should clarify whether the variable t represents time or iteration number and ensure consistency throughout the section.

Answer 1. Thank you for your valuable suggestions. The original formula (23) has been supplemented and improved to become formula (25). The variable $t$ represents the iteration time. After unanimous discussion among the authors, we have made the entire part consistent so that the variable $t$ in the entire part has the same meaning. This will help the authors have a deeper understanding of this part.

2. Cutoff condition: The authors should provide a clear definition and explanation of the "cutoff condition" in the context of elasticity optimization strength control and how it relates to the optimization process.

Answer 2. This chapter describes the specific steps of the elastic firefly algorithm for optimizing PID parameters, and further explains and defines the concept of "cutoff condition". The meaning of "cutoff condition" is "reaching the maximum number of iterations or the ratio of high-likelihood particles is greater than the maximum ratio $\text{pr}_{\text{max}}$". The role of the "cutoff condition" is to directly limit the running time of the algorithm to avoid infinite loops. Too many iterations may result in too high a time cost, and too few iterations may result in failure to find the optimal solution. If the solution found by the algorithm reaches this goal, the optimization is considered successful and the optimization process can be terminated in advance. This improvement will give the author a clearer understanding of this optimization step.

3. Equation (32): The authors should offer guidance on selecting an appropriate value for the constant c and discuss its impact on the optimization process.

Answer 3. The original formula (32) has been supplemented and improved to become formula (34). The characteristics of the particle density detection problem are analyzed, including the size of the search space, the complexity of the objective function, whether there are multiple local optimal solutions, etc., and appropriate constants are selected. Multiple experiments are conducted, and different $c$ values are used to observe the optimization effect of the algorithm, including the convergence speed and the quality of the optimal solution. When searching, a larger $c$ value is used to speed up the global search speed. As the number of iterations increases, the $c$ value is gradually reduced to improve the accuracy of the local search. Through experimental tuning, the optimal $c$ value is gradually determined. For problems with a large search space and multiple local optimal solutions, a larger value can be selected to increase the search range and improve the global search capability. In this chapter, the constant is an important indicator that affects the rebound coefficient. $\frac{c}{k_{s}}$ is a constant. The value of $\frac{c}{k_{s}}$ is related to the rebound coefficient. The influence of the value of $\frac{c}{k_{s}}$ can be eliminated. Therefore, for the convenience of calculation, the rebound coefficient can be directly taken as 1.

Too large or too small $c$ values will have different effects on the particle density detection process. A larger c value: increases the rebound distance of the firefly, a wider search range, helps to jump out of the local optimal solution, and increases the possibility of global search. Reducing the rebound distance of fireflies and making the search more precise helps to find better solutions in local areas, but may lead to local optimality.

REVIEW OF SECTION 3.1.1:

The authors introduce three key concepts in this section: particle fluorescence brightness, particle attraction, and particle position update. They provide equations and explanations for each concept, which are generally clear and consistent with the principles of the FA algorithm.

Particle Fluorescence Brightness:

Equation (23) defines the particle fluorescence brightness Iⁿᵢ(t) as a measure of the particle's position, with higher values indicating better positions. The equation is consistent with the concept of fluorescence brightness in the FA algorithm.

Comment: The authors use inconsistent notation for the particle index, denoting it as "i" in Equation (23) and "ρ" in subsequent equations and explanations. They should maintain consistent notation throughout the section to avoid confusion.

Answer: The original formula (23) has been supplemented and improved to become formula (25). We have unified the symbols of particle indices in subsequent formulas into "$i$"/"$I$" to avoid confusion and provide readers with clearer interpretation and understanding.

Particle Attraction:

Equation (25) defines the attraction of the best particle ρmax to particle ρ, which is consistent with the concept of attraction in the FA algorithm.

Comment: The authors use inconsistent notation for the distance between particles, denoting it as "rᵢmax(t)" in Equation (25) and referring to it as "rⁿᵢmax(t)" in the explanation. They should clarify the correct notation and ensure consistency throughout the section.

Answer: The original formula (25) has been supplemented and improved to become formula (27). After joint review by the authors, the symbols in formula (27) have been unified and improved. "$r_{\text{i}\gamma_{\max}n(t)}$" is used in both the formula and the annotation. This will ensure the uniformity of the entire formula.

Particle Position Update:

Equation (26) describes the process of updating particle positions based on the attraction of the best particle and a random component, which is consistent with the concept of particle movement in the FA algorithm.

Areas requiring further clarification:

1. Gain coefficients: The authors should provide guidance on selecting appropriate values for the gain coefficients α₁ and α₂ and discuss their impact on the optimization process.

Answer 1. The gain coefficient $\omega_{1}$ is usually used to adjust the influence of the optimal particle, that is, the weighting coefficient when a particle (or firefly) moves towards the optimal solution.

(1) For problems with a large search space and high complexity, a larger $\omega_{1}$ value can be selected to enhance the attraction of the optimal particle so that it can guide other particles toward the optimal solution more quickly. A larger $\omega_{1}$ value helps to develop and quickly concentrate near the current optimal solution.

(2) For simpler problems, a smaller $\omega_{1}$ value may be more appropriate to prevent premature convergence to the local optimal solution. A smaller $\omega_{1}$ value helps to explore and conduct a wider search in the search space.

However, the optimal value is not fixed and may need to be determined through experimentation and tuning. Some methods include:

Conduct a series of experiments with different $\omega_{1}$ values to observe the performance of the algorithm on a specific problem.

Consider the characteristics of the problem, such as the complexity of the problem, the size and shape of the search space, etc. For complex multi-peak problems, a larger $\omega_{1}$ value may be required to avoid premature convergence to the local optimum. Combined with the adjustment of other parameters, such as inertia weight, to balance global and local search capabilities.

In this article, the initial value of $\omega_{1}$ can usually be set between 0.5 and 1.0. Values in this range can balance the development and exploration of the algorithm. For problems with complex search spaces and multiple local optimal solutions optimized by the elastic FA algorithm, a larger $\omega_{1}$ value (e.g., 0.8 to 1.0) can be selected to enhance the attraction to the global optimal solution and quickly guide the fireflies to the optimal solution. Conduct multiple experiments, each using different $\omega_{1}$ values, observe the convergence speed and the quality of the solution, and select the optimal $\omega_{1}$ value.

The gain coefficient $\omega_{2}$ is usually used to adjust the influence of the random function, that is, the random perturbation or random search intensity in the algorithm.

(1) For problems with multiple local optimal solutions, a larger $\omega_{2}$ value can increase the randomness of the search and help to jump out of the local optimal solution. A larger $\omega_{2}$ value may cause instability in the early stages of the algorithm, but it helps global search.

(2) For smooth objective functions, a smaller $\omega_{2}$ value can make the search process more stable and reduce unnecessary random perturbations. A smaller $\omega_{2}$ value can improve the stability of the algorithm and is suitable for later fine-grained search.

Different $\omega_{2}$ values can be tested through experimental tuning methods to observe their impact on the performance of the algorithm and select the $\omega_{2}$ value with the best performance.

Since it can provide a moderate amount of randomness in the early stages, it can balance exploration and development. In the early stages of the algorithm, more randomness is needed to explore the search space; in the later stages, less randomness is needed for fine optimization. You can set the $\omega_{2}$ value between 0.1 and 0.5, run the algorithm multiple times, and adjust the $\omega_{2}$ value each time (for example, from 0.1 to 0.5 in steps of 0.1), and record the optimization results of each experiment, including the convergence speed, the quality of the optimal solution, and the stability of the algorithm. Compare the experimental results under different $\omega_{2}$ values to find the $\omega_{2}$ value with the best performance.

$\omega_{2}$ controls the speed and degree of the particle's approach to the optimal particle (that is, the currently known optimal solution). It determines the development ability, that is, the ability to conduct a fine search near the known optimal solution.

$\omega_{2}$ controls the intensity of random perturbations and increases the randomness of the algorithm. It determines the exploration ability, that is, the ability to conduct a wide search in the search space.

2. Random function: The authors should clarify the range of the uniform distribution satisfied by the random function "rand" to ensure reproducibility.

Answer 2. The random function $\text{rand}$ in this part satisfies the standard uniform distribution and usually takes values in the range [0,1). This means that the random numbers generated by $\text{rand}$ are between 0 and 1 (including 0 but excluding 1), and the probability of each number appearing is the same. The following is the formula for the random number $\text{rand}$.

$$\text{rand}_{[-a,a)} =2a\cdot(\text{rand}-0.5)$$

3. Convergence properties and termination criteria: The authors should provide insight into how the algorithm determines when to stop updating particle positions and consider the solution converged.

Answer 3. The original formula (26) has been supplemented and improved to become formula (28). We set a maximum number of iterations before the algorithm starts. When the number of iterations reaches this predetermined value, the algorithm stops. A threshold is set. When the change in the objective function value in several consecutive iterations is less than the set threshold, it is considered to have converged.

REVIEW OF SECTION 3.1.2:

The authors introduce the concept of elasticity optimization strength control to prevent unreasonable particle aggregation and over-optimization, which can lead to computational burden and low-quality convergence. They propose using the proportion of high-likelihood particles, the maximum number of iterations, and a particle density detection mechanism to control the optimization intensity.

Particle Density:

Equation (27) defines particle density ρcr(m) as the number of particles within a specified judgment radius rjudg around the optimal particle ρmax(n). The equation is consistent with the concept of particle density and provides a quantitative measure of particle aggregation.

Comment: The authors use inconsistent notation for the particle index, denoting it as "i" in Equation (27) and "ρ" in the previous section. They should maintain consistent notation throughout the paper to avoid confusion.

Answer: The original formula (27) has been supplemented and improved to become formula (29). After joint review by the authors, the symbols in formula (29) have been unified and improved. We have unified the symbols of particle indices in other formulas into "$i$"/"$I$" to avoid confusion and provide readers with clearer interpretation and understanding.

Ratio of High-Probability Particles:

Equation (28) defines the ratio of high-likelihood particles pr(m) as the ratio of the particle density ρcr(m) to the total number of particles N. The equation is consistent with the concept of measuring the proportion of particles that are likely to be close to the optimal solution.

Area requiring further clarification: The authors should provide a clear threshold or criterion for determining which particles are considered high-likelihood to ensure that the ratio of high-likelihood particles is a meaningful measure of optimization progress.

Answer: High probability particles usually refer to those that perform well in the current iteration, that is, particles with low objective function values. In terms of setting thresholds, commonly used methods include fixed thresholds, dynamic thresholds based on mean and standard deviation, and thresholds based on percentiles. The criteria and thresholds for selecting high probability particles can be based on weight thresholds, normalized weights, percentiles, fixed thresholds, and standard deviations. In this chapter, the author selects particles with weights in the top 10% to 20% as high probability particles. For example, if there are 1,000 particles, the top 100 to 200 particles are selected as high probability particles. This method can help the filter focus more effectively on the most likely area in the state space and improve the accuracy of state estimation. And improve the efficiency of the filter. However, this method needs to be adjusted according to the specific application to ensure that important state information is not lost while retaining high probability information.

Optimization Strength Control:

The authors propose controlling the optimization strength by combining the ratio of high-likelihood particles pr(m) with the maximum number of iterations. They suggest stopping the optimization process when the ratio of high-likelihood particles in the nth iteration exceeds a set threshold prmax or when the number of iterations exceeds the maximum number of iterations.

Areas requiring further clarification:

1. Selection of prmax: The authors should discuss the factors that influence the choice of the maximum ratio of high-likelihood particles and provide recommendations based on their experience or empirical results.

Answer 1. In the optimization process of the elastic firefly algorithm, the selection of the maximum ratio $\text{pr}_{\text{max}}$ of high-likelihood particles is a key factor. It directly affects the convergence speed, global search capability and final optimization effect of the algorithm. According to the complexity of the optimization problem, the dimension, the number of particles, the number of iterations and the quality of the initial solution layout, different maximum ratios $\text{pr}_{\text{max}}$ of high-likelihood particles are selected, and references are cited in the article to prove it.

The selection of the maximum ratio $\text{pr}_{\text{max}}$ of high-likelihood particles is affected by multiple factors, including the complexity of the problem, the number of particles, the number of iterations and the distribution of the initial solution. Based on the rule of thumb and empirical results, the initial value can be selected between 10% and 30%, and this ratio can be dynamically adjusted during the optimization process. By reasonably selecting and adjusting the high-likelihood particle ratio, the convergence speed and global search capability of the algorithm can be effectively balanced to ensure the effectiveness and efficiency of the optimization process.

2. Trade-off between prmax and maximum number of iterations: The authors should provide insight into how these two criteria interact and affect the optimization process, and how to balance them for effective optimization strength control.

Answer 2. In the optimization algorithm, the maximum ratio $\text{pr}_{\text{max}}$ of high-likelihood particles and the maximum number of iterations are two key parameters. They interact with each other and jointly affect the efficiency and effect of the optimization process. They are essential for effectively controlling the optimization intensity, improving the convergence speed, and finding the global optimal solution. Through gradual $\text{pr}_{\text{max}}$, adaptive number of iterations, and empirical rules, a balance can be found between maintaining global search capabilities and accelerating convergence to effectively control the optimization intensity and ensure that the optimal solution is found within reasonable computing resources.

3. Premature convergence: The authors should discuss strategies to mitigate the risk of premature convergence, such as incorporating a minimum number of iterations or a diversity maintenance mechanism.

Answer 3. Premature convergence is a common problem in optimization algorithms, especially when dealing with complex, multi-peak objective functions. Premature convergence means that the algorithm stagnates in a local area of the search space and fails to fully explore the entire space, thus missing the global optimal solution. To mitigate the risk of premature convergence, the following strategies can be adopted:

Minimum number of iterations:

The minimum number of iterations refers to the minimum number of iterations enforced during the optimization process. Even if a better solution has been found, the algorithm must continue to execute until this number of iterations is reached. Set a minimum number of iterations and stop iterating when the change in the objective function is less than the set threshold. If the algorithm does not meet the convergence criterion within the minimum number of iterations, continue to iterate. Determine whether to continue iterating by evaluating the quality and improvement of the current solution. This ensures that the algorithm has enough time to explore the search space. Add a conditional judgment to the algorithm to ensure that the convergence judgment is made after the number of iterations reaches the minimum requirement.

Diversity maintenance mechanism:

The diversity maintenance mechanism is a key strategy in optimization algorithms, which aims to maintain the diversity of the solution space and prevent the algorithm from converging to the local optimal solution prematurely. By introducing a diversity maintenance mechanism, the global search ability of the algorithm can be improved, so as to find a better solution. Through adaptive mutation, elite retention, group diversity indicators, population splitting, random restart and diversity promotion mechanisms, the diversity of the solution space can be effectively maintained and the possibility of finding the global optimal solution can be increased. Reasonable design and implementation of these mechanisms will significantly improve the performance and reliability of the optimization algorithm.

Your review of section 3.1.3 "Particle Density Detection" is thorough and well-organized. You have identified several inconsistencies and areas that require further clarification, which will help improve the scientific rigor and practicality of the proposed approach. Let's summarize the key points and provide a comprehensive review of the section.

REVIEW OF SECTION 3.1.3:

The authors introduce a particle density detection mechanism to measure the particle distribution after iteration and adjust the particle distribution around the optimal particle if the density exceeds a specified threshold. They propose constructing a spring system between the particles in the judgment area and the optimal particle, with the compression of the spring equal to the distance the particle moves towards the optimal particle.

Rebound Coefficient:

Equation (31) defines the rebound coefficient cⁿᵢ as the ratio of the elastic force Fⁿᵢ to an unspecified parameter β. The equation is consistent with the concept of a rebound coefficient, which determines the magnitude of the particle's rebound when the density is too high.

Inconsistencies and areas requiring further clarification:

1. Definition of β: The authors should clarify the meaning of β and its role in the rebound coefficient calculation.

Answer 1. Thank you for your valuable comments on this section. We will make unified revisions and improvements to this section based on all your comments above. The original formula (31) has been supplemented and improved to formula (33). $\beta$ should be the constant $c$ in formula (33). Constant $c$ is an adjustment factor used to standardize the calculation of the coefficient of resilience to ensure that the result conforms to a specific dimension or unit. It ensures the consistency of the coefficient of resilience in the calculation and makes its value applicable to different calculation scenarios. It can help to make a unified comparison under different physical conditions or different measurement units. Its function is to adjust the coefficient of resilience.

2. Relationship between Equations (31) and (32): The authors should explain how the alternative expression for the rebound coefficient cⁿᵢ in Equation (32) is derived from Equation (31) and how it relates to the previous definition.

Answer 2. The original formulas (31) and (32) have been supplemented and improved to become formulas (33) and (34). The alternative expression for the coefficient of resilience $\zeta$ (34) is to substitute the elastic force $F_{n}^{i}$ in formula (32) into formula (33), and then simplify and organize the form to obtain formula (34). The relationship between the coefficient of resilience and the previous one is that they both involve the behavior and characteristics of objects or particles when they collide or interact. This relationship has wide applications in physics and materials science, especially in studying the behavior of granular materials, powders, fluids, and solid surfaces. Materials of different densities have different rebound characteristics. Materials with higher density usually have lower coefficients of resilience because they lose more energy when they collide. The coefficient of resilience can be used to understand the collision behavior of granular materials, which helps to infer their density distribution. For example, during the flow and accumulation of granular materials, collision behavior (such as elastic and inelastic collisions) affects the density distribution. This relationship has wide applications in fields such as materials science, soil mechanics, and fluid mechanics, and helps to optimize material properties, evaluate slope stability, and improve multiphase flow models.

3. Selection of constant c: The authors should provide guidance on how to select an appropriate value for the constant c and discuss its impact on the particle distribution adjustment process.

Answer 3. In some cases, a constant $c$ may be introduced into the formula of the coefficient of rebound to adjust the coefficient of rebound. The selection principle of this constant $c$ mainly depends on the specific experimental conditions and material properties. By measuring equipment errors, considering the influence of environmental factors, reflecting material properties, fitting experimental data and referring to industry standards, the value of constant $c$ can be determined to better adapt to specific experimental conditions and material properties. This is of great significance for improving the reliability and repeatability of detection results.

Constant $c$ can be used to compensate for the energy loss between particles and particle surfaces during the collision. Different particle distributions will lead to different energy losses. Adjusting $c$ can help more accurately reflect the actual energy transfer and loss. By adjusting $c$ , the velocity distribution of particles after the collision can be changed, which in turn affects the measurement results of particle distribution.

Update of Particle Position under the Action of an Elastic Mechanism:

Equation (33) describes the process of updating particle positions when the particle density exceeds the specified threshold. The particles in the judgment area should rebound in the opposite direction of compression, with the rebound distance determined by the rebound coefficient.

Areas requiring further clarification:

1. Threshold value for particle density: The authors should provide a clear criterion for determining when the particle density is considered too high and requires adjustment.

Answer 1. Thanks to the experts for their valuable suggestions. The original formula (33) has been supplemented and improved to formula (35). In particle density detection, determining when the particle density is too high and needs to be adjusted is a key step. The setting of the particle density threshold is a process that comprehensively considers application requirements, equipment performance, experimental data, safe operation and other factors. Through clear standards and methods, it can be ensured that the adjustment of particle density is timely and effective to maintain the optimal performance and safety of the system. The threshold is set and adjusted according to actual conditions to adapt to different application environments and requirements.

The number and distribution of particles in the system will affect the setting of the density threshold. For example, if there are more particles and they are evenly distributed, the threshold can be set higher; if there are fewer particles and they are unevenly distributed, a lower threshold can be set to capture local high-density areas.

2. Impact on convergence properties: The authors should provide insight into how the particle position adjustment affects the overall optimization process and whether it may lead to instability or oscillations in certain cases.

Answer 2. Thanks to the experts for their valuable opinions, updating the position adjustment of rebound particles can significantly affect the optimization process. Through the rebound mechanism, particles can quickly get rid of the local optimal solution, thereby speeding up the convergence to the global optimal solution. The rebound mechanism can help particles re-explore the search space, thereby improving the exploration capability of the algorithm. The rebound mechanism enables particles to jump out of the current local dense area, explore a larger search space, and increase the probability of finding the global optimal solution. In a dynamic or complex search space, the rebound mechanism can enhance the adaptability of the algorithm, making it better able to cope with changes and diversity in the search space.

If the rebound coefficient is not chosen properly, particles may rebound excessively, causing large oscillations during the search. Excessive rebound may cause the particles to move away from the current optimal solution, increase the convergence time, or fall into oscillation, making it impossible to stabilize near the optimal solution. If the rebound coefficient is too small and the rebound effect is not obvious, the particles may not be able to effectively jump out of the local optimal solution, thus reducing the optimization efficiency. In some complex optimization problems, the appropriate rebound coefficient may need to be dynamically adjusted. The fixed rebound coefficient cannot cope with the diversity and complexity of the search space, which may lead to instability or oscillation. And improved the reference materials of this part.

3. Computational complexity: The authors should discuss the scalability of the proposed approach, especially for high-dimensional optimization problems or large particle populations.

Answer 3. The FA optimization principle of the elastic mechanism has good scalability to a certain extent, especially in the case of high-dimensional optimization problems or large particle swarms. This mechanism can effectively improve the algorithm's search ability and convergence performance. However, as the problem dimension increases and the size of the particle swarm expands, it also brings some challenges and factors that need to be considered.

The complexity of the search space of high-dimensional optimization problems grows exponentially, which increases the difficulty of the algorithm to find the global optimal solution. The elastic mechanism can alleviate this problem to a certain extent by increasing the exploration ability of particles.

Particles in high-dimensional space may require a larger rebound coefficient to effectively explore the entire space. However, an excessively large rebound coefficient may cause particle oscillation and instability. An adaptive rebound coefficient adjustment strategy needs to be designed to balance the relationship between exploration and development.

The computational complexity of high-dimensional optimization problems is high, especially when calculating particle density and interaction forces. It is possible to consider introducing sparse representation or dimensionality reduction techniques to reduce computational complexity.

Large particle swarms can increase the coverage of the search space and increase the probability of finding the global optimal solution. However, the interaction computation of large-scale particle swarms is large, which may significantly increase the running time of the algorithm. Efficient parallel computing and distributed computing technologies are needed to accelerate the calculation of particle interactions.

Large particle groups are more likely to fall into local optimal solutions. The introduction of elastic mechanisms can help particles jump out of local optimal solutions, but the rebound mechanism also needs to be reasonably adjusted to avoid frequent invalid jumps and oscillations. And the reference materials for this part are improved.

SECTION 3.2: PARTICLE WEIGHT FILTER COMPENSATION CONTROL DESIGN

The authors introduce the concept of necessary sampling and propose a weight compensation function to ensure the uniformity of the weighted particle distribution before and after optimization. The equations and explanations are generally clear and consistent with the principles of particle filtering and importance sampling.

Answer. Thank you for your careful review and positive comments on the importance density function in our paper. Your feedback is of great significance for us to further improve our work. Thank you for your affirmation of the weight compensation function we proposed. The weight compensation function is designed to ensure the uniformity of the weighted particle distribution before and after optimization. This design is based on the principles of particle filtering and importance sampling, and aims to improve the representativeness of the sample set and the accuracy of the final result. We are committed to providing clear and detailed equations and explanations in the paper to ensure that readers can fully understand our methods and principles.

However, there are a few points that require further clarification:

1. Integration of the exponentially convergent disturbance observer: The authors should provide a clear explanation of how this observer is integrated into the overall control scheme and how it interacts with the particle weight filter compensation.

Answer 1. The Exponential Convergence Disturbance Observer is a tool for dynamic systems designed to estimate and compensate for disturbances in the system in real time. Integrating it into the control design scheme after particle weight filter compensation can significantly improve the robustness and stability of the system. Particle filters can provide accurate system state estimates through particle weight updating and resampling. However, the accuracy of state estimation may decrease when unknown disturbances are present in the system. By integrating a disturbance observer, unknown disturbances in the system can be estimated and compensated in real time, thereby improving the accuracy of particle filter state estimation.

There is a tight feedback loop between the particle filter and the interference observer. The particle filter provides a system state estimate, and the disturbance observer adjusts the disturbance estimate based on this state estimate, thereby improving control accuracy.

Through the dual mechanism of weight compensation and interference observer, the dynamic response and anti-interference ability of the system can be effectively improved. And improved the reference materials of this part.

2. Equation (44): The authors should discuss how the gains kp, ki, and kd are tuned or optimized using the elastic FA algorithm and provide more details on the integration of the FA algorithm with the PID controller tuning process.

Answer 2. Using the elastic firefly algorithm (FA) to optimize the PID gain parameters ($k_{\text{P}}$, $k_{\text{I}}$ and $k_{\text{D}}$) can significantly improve the performance of the control system, thereby improving the performance of the control system. Optimizing the gain parameters of the PID controller requires defining an objective function, which is usually based on the performance indicators of the control system, such as steady-state error, rise time, overshoot, and adjustment time. Commonly used objective functions include integrated absolute error (IAE), integrated squared error (ISE), integrated time absolute error (ITAE), etc. Then perform the initialization, brightness calculation, position update, and iterative optimization of the FA algorithm. When searching for optimal PID parameters, real-time estimation and compensation of disturbances are considered, making the control system more robust and stable in the face of external disturbances.

The following are the key steps in the process of integrating the elastic FA algorithm and the PID controller based on the disturbance observer for gain parameter optimization:

(1) Establish a system model, including dynamic equations and disturbance observers.

(2) Define the objective function based on the control system performance indicators.

(3) Initialize FA algorithm parameters and firefly population.

(4) Through the iterative optimization process, gradually adjust the firefly position to find the optimal PID parameter combination.

(5) Use the optimized PID parameters to conduct system simulation to verify and analyze the controller performance.

This integrated approach not only improves control accuracy but also maintains system stability in complex dynamic environments.

Your review of section 3.2 "Particle weight filter compensation control design" is comprehensive and well-structured. You have identified several inconsistencies and areas that require further clarification, which will help improve the scientific rigor and practicality of the proposed approach. Let's summarize the key points and provide a comprehensive review of the section.

Importance Density Function:

The authors define the importance density function g[x(t)] as the probability density function from which the optimized sample set {xⁿᵢ(t)} is drawn, as opposed to the desired posterior probability density function p[x(t)|Z(t-1)]. This concept is consistent with the principles of importance sampling and provides a basis for the weight compensation process.

Answer. We choose to define $g[x(t)]$ as the probability density function for extracting the optimized sample set, mainly to simplify the calculation process and improve the efficiency of the algorithm. The posterior probability density function $I[x\left(t\right)\text{∣}Z(t-1)]$ may be difficult to obtain or calculate directly in practical applications, so sampling through the importance density function $g[x(t)]$ is a common and effective alternative. This approach is consistent with the principle of importance sampling and provides the necessary foundation for the subsequent weight compensation process to ensure that readers can fully understand our method. Thank you for your careful review and valuable suggestions, which will help us further refine and improve our work. If you have any further comments or suggestions, we are very happy to listen and make corresponding improvements.

Weight Compensation Function:

Equation (35) proposes a weight compensation function to ensure the uniformity of the weighted particle distribution before and after optimization. The function is based on the ratio of the posterior probability density function to the importance density function, as shown in Equation (34).

Inconsistencies and areas requiring further clarification:

1. Relationship between R(t)ⁱ and ωⁱ(t): The authors should explain the relationship between the ratio of the posterior probability density function to the importance density function, denoted as R(t)ⁱ in Equation (34), and the weight compensation function, denoted as ωⁱ(t) in Equation (35).

Answer 1. The original formulas (34) and (35) have been supplemented and improved, and are now formulas (36) and (37). The relationship between formulas (36) and (37) is to convert the weight $R^{i}\left(t\right)$ in formula (36) Substituted into the weight compensation function of the optimized particle in formula (37), the two have a nested relationship. Weights are of great significance in optimizing the weight compensation function of particles. It reflects the credibility of each particle, affects the optimization direction, guides the particle swarm to approach the optimal solution, while maintaining the diversity of the particle swarm and avoiding falling into local optimality. By properly calculating and using weights, the performance and stability of particle filtering and optimization algorithms can be significantly improved.

2. Estimation of density functions: The authors should discuss the computational aspects of evaluating the posterior probability density function p[x(t)|Z(t-1)] and the importance density function g[x(t)] in practice, as well as the potential challenges or limitations involved.

Answer 2. Thanks to the reviewers for their valuable comments, I continued to improve and perfect the calculations in this part, unified the symbolic expressions, and related $g[x(t)]$ more closely to $I[x\left(t\right)\text{∣}Z(t-1)]$. Due to the length and simplicity of the article, the following is Responses elaborated on potential challenges or limitations:

High-dimensional problems: In high-dimensional state spaces, the computational complexity of posterior probabilities increases exponentially, resulting in significant increases in computing resources and time requirements. Nonlinear and non-Gaussian systems: For nonlinear and non-Gaussian systems, traditional analytical methods are difficult to effectively calculate the posterior probability, and numerical approximation methods, such as particle filtering, are needed. Computing resources: Real-time computing has high requirements and requires a large amount of computing resources and optimization algorithms to ensure real-time performance.

3. Integration with particle filter algorithm: The authors should provide a more detailed description of the complete particle filter algorithm with weight compensation and clarify the sequence of steps involved, including how the weight compensation function interacts with the resampling step.

Answer 3. The complete particle filter algorithm with weight compensation is a recursive estimation process for state estimation of dynamic systems. The algorithm iterates through prediction, update, and resampling steps to ensure the diversity of particles and the accuracy of state estimation. The steps of the complete particle filter algorithm with weight compensation are as follows:

(1) Initialization: Initialize the initial positions and weights of the particles. The positions of the particles are randomly distributed in two-dimensional space, and the weights are equally distributed.

(2) State prediction: Use the state transition model to predict the next moment position of the particle to simulate the dynamic changes of the system.

(3) Update weight: Calculate the weight of each particle according to the observation model. The weight reflects the degree of match between the particle and the actual observation value.

(4) Normalize weight: Ensure that the sum of the weights is 1 to avoid error accumulation in numerical calculations.

(5) Weight compensation: Determine whether resampling is needed by calculating the number of effective samples. If Neff is lower than the threshold, resampling is performed to maintain the diversity of the particle population.

(6) Resampling: Resample particles according to weights to avoid weight degradation and maintain the diversity of the particle swarm.

(7) State estimation: Calculate the estimated value of the system state based on the resampled particles, usually taking the mean of the particle positions.

(8) Weight reset: If resampling is performed, reset the weights to be evenly distributed to prepare for the next iteration.

Through the above steps, the particle filter algorithm with weight compensation can gradually approach the true state in a dynamic system and provide reliable state estimation. Weight compensation and resampling steps are key links to ensure the stability and performance of the algorithm.

Exponentially Convergent Disturbance Observer:

Equation (36) introduces an exponentially convergent disturbance observer to estimate the system disturbance and compensate for it in the PID controller. The observer gain K is chosen to ensure exponential convergence of the estimation error.

Areas requiring further clarification:

1. Selection of observer gain K: The authors should provide guidance on how to select an appropriate value for the observer gain K and discuss the trade-off between convergence speed and noise sensitivity.

Answer 1. Thanks to the experts for their valuable review comments. The original formula (36) has been supplemented and improved to formula (38). Due to the length of the article, a detailed answer to this question is given below.

The disturbance observer is a technology used to estimate and compensate for system disturbances. In the design of the disturbance observer, the disturbance convergence coefficient $K$ determines the convergence speed of the disturbance estimation and the rapidity of the system response. Selecting a suitable $K$ value is crucial to ensuring the stability and performance of the system. Several parameters that need to be mainly referenced are system characteristic analysis, frequency domain analysis, simulation and experimental adjustment, etc. A larger $K$ value will cause the disturbance observer to respond to system disturbances faster, but may introduce high-frequency noise. A smaller $K$ value will cause the disturbance observer to respond to disturbances more smoothly and reduce the impact of high-frequency noise, but the response speed will be slower. Through frequency response analysis, the value of $K$ can be determined so that the disturbance observer has good estimation performance within a specific frequency range. In the design process, $K$ can be selected so that it has high gain in the low frequency band (the frequency band where the disturbance mainly exists) and low gain in the high frequency band to suppress noise. When the $K$ value is initially selected, it can be based on experience or the aforementioned analysis method. Through simulation and experiment, adjust the $K$ value, observe the disturbance estimation effect and control performance of the system, and select the optimal $K$ value.

The disturbance convergence coefficient $K$ plays a key role in the design of the disturbance observer. It directly affects the convergence speed of the disturbance estimation and the sensitivity to noise. Selecting a suitable $K$ value is the key to ensuring the stability and performance of the system. It is usually necessary to find a balance between the convergence speed and noise sensitivity. In the design of the disturbance observer, selecting a suitable disturbance convergence coefficient $K$ requires finding a balance between the convergence speed and noise sensitivity. A larger $K$ value can increase the convergence speed, but increase the sensitivity to high-frequency noise; a smaller $K$ value can reduce the noise sensitivity, but slow down the convergence speed. Through system dynamic analysis, frequency domain analysis, and simulation and experimental verification, the optimal $K$ value can be determined to achieve the best performance of the system.

2. Robustness to uncertainties and noise: The authors should analyze the sensitivity of the disturbance observer to model uncertainties and measurement noise, and propose strategies to mitigate their impact on the estimation accuracy.

Answer 2. Thank you for the valuable review comments from the experts. Due to the length of the article, a detailed answer to this question is given below.

Uncertainty refers to the deviation between the actual system and the design model. These deviations may come from changes in system parameters, model simplification, and unmodeled dynamics. Model uncertainty will increase the estimation error of the disturbance observer, thereby affecting the control performance of the system. If the disturbance observer relies on inaccurate model information, its estimated disturbance may deviate from the actual disturbance, resulting in unsatisfactory compensation effect.

Measurement noise refers to the random error or interference signal introduced during the sensor measurement process. Measurement noise will cause the input signal of the disturbance observer to contain noise components, which will lead to inaccurate disturbance estimation. High-frequency noise is particularly easy to amplify through the transfer function of the disturbance observer, causing high-frequency oscillation of the estimated signal.

In practical applications, the disturbance observer is greatly affected by model uncertainty and measurement noise. Through robust design, adaptive algorithms, hybrid control strategies, low-pass filtering and Kalman filtering, the impact of these factors on the estimation accuracy can be effectively reduced and the overall performance of the system can be improved.

References are cited for evidence.

3. Integration with PID controller: In Figure 4, the authors should clarify how the disturbance estimate from the observer is integrated into the PID controller and how it affects the control signal.

Answer 3. Thank you for the valuable review comments from the experts. Due to the length of the article, a detailed answer to this question is given below.

In Figure 4, this is a specific control system design, which includes a disturbance observer, a PID controller, and the execution part of the system. This design details how the disturbance observer is integrated into the PID controller and how it affects the control signal. Integrating the disturbance observer into the PID controller is an effective way to improve the robustness and performance of the control system. The disturbance observer can estimate the disturbances in the system and use the estimated values of these disturbances to compensate the control signal, thereby improving the control system's ability to resist disturbances. The following details how the disturbance observer is integrated into the PID controller and explains its impact on the control signal. The disturbance observer is integrated into the PID controller through the steps of error signal generation, PID controller, disturbance compensation, system response, closed-loop feedback, etc.

(1) Error signal generation:

The error signal $e$ is generated by the difference between the expected output $y_{d}$ and the actual output $y$. The error signal is the input of the PID controller and is used to adjust the control output.

(2) PID controller:

The PID controller calculates a control command $u$ based on the error signal. The control command $u$ is designed to adjust the system to reduce the error and move closer to the desired output.

(3) Integration of disturbance observer:

The disturbance observer estimates the external disturbance $\hat{d}$ based on the system output $y$, the derivative of the output , and the system input $u$. The disturbance estimate $\hat{d}$ is calculated from , where $a_{1}$ and $a_{2}$ may be parameters based on the system dynamics.

(4) Disturbance compensation:

The estimated disturbance $\hat{d}$ is used to directly adjust the control command $u$ of the PID controller as a compensation term. Such adjustments help to reduce the impact of external disturbances and improve the stability and accuracy of the system.

(5) System response:

The adjusted control command $u$ acts on the system through the system dynamics (such as $1/b$ in the figure represents a certain gain or dynamic characteristic of the system) to produce the actual output $y$.

(6) Closed-loop feedback:

The system output $y$ is fed back to the error calculation and disturbance observer. This closed-loop structure ensures that the system can continuously self-correct in response to external changes and dynamic changes within the system.

Through this design, the integration of the disturbance observer not only helps the system to identify and compensate for external disturbances, but also enhances the effect of the PID controller, making the entire control system more robust and effective.

Comments in Figure 4:

The block diagram of the elastic FA algorithm optimized filter adaptive PID control structure in Figure 4 shows the disturbance observer as a separate block without any connections to the other components of the control system. The authors should revise the figure to clearly illustrate how the disturbance estimate is integrated into the PID controller and how it interacts with the other elements of the control structure.

Answer: Thank you for your valuable comments on Figure 4. We will modify and improve this part based on all your comments above. The author has added a connection line from the interference observer to the PID control module to provide a better interface for the interference observer. "Interference compensation" of the PID control module, and add the symbols of positive and negative feedback to the sensor in the figure: "+" and "-". This can more clearly explain the significance of this figure and pave the way for introducing the steps of the optimization algorithm. The following is the improved Figure 4. If there is something wrong, please ask experts to give their opinions and we will definitely modify it:

SECTION 3.3: OPTIMIZING PID PARAMETERS USING THE ELASTIC FA ALGORITHM

The authors provide a step-by-step procedure for applying the elastic FA algorithm to tune the PID gains. The optimization steps are clearly explained and consistent with the principles of the elastic FA algorithm introduced earlier.

HOWEVER, there are a few areas that could benefit from further discussion:

1. Computational complexity and convergence properties: The authors should discuss the algorithm's efficiency and scalability, especially for real-time control applications.

Answer 1. Thanks to the experts for their valuable opinions on this part. After unanimous discussion among our authors, this part supplements and improves the efficiency and reliability of the elastic FA algorithm optimized filtering adaptive PID control structure of the hydrostatic drive system of mining concrete mixing tank trucks. Discussion on scalability, with special emphasis on real-time control applications, "The elastic FA algorithm can globally optimize PID control parameters to ensure that the system can maintain optimal performance under various working conditions. By adjusting control parameters in real time, the system can Achieve more precise control and reduce overshoot and oscillation phenomena. The elastic FA algorithm has good global search capabilities and adaptability, and can find optimal control parameters for the system under different working conditions, whether under high load or low load conditions. It can maintain efficient operation through adaptive adjustment. At the same time, its strong adaptability, modular design, real-time performance and integration enable it to maintain efficient and stable operation under various complex working conditions, especially in real-time control applications. It can significantly improve the response speed, control accuracy and stability of the system. "The purpose of this is to allow readers to better understand the innovations described in this article.

The elastic FA algorithm can globally optimize PID control parameters to ensure that the system can maintain optimal performance under various working conditions. By adjusting control parameters in real time, the system can achieve more precise control and reduce overshoot and oscillation. The elastic FA algorithm has good global search capabilities and adaptability, and can find optimal control parameters under different working conditions. Whether under high or low load conditions, the system can maintain efficient operation through adaptive adjustment. At the same time, its strong adaptability, modular design, real-time and integration enable it to maintain efficient and stable operation under various complex working conditions. Especially in real-time control applications, it can significantly improve the response speed and control of the system. Accuracy and stability.

2. Comparison with other optimization algorithms and adaptive control techniques: Including such a comparison would help highlight the advantages and limitations of the elastic FA algorithm optimization filter for PID controller tuning.

The authors propose using the elastic firefly algorithm to optimize the PID controller parameters (kp, ki, and kd). They provide a step-by-step procedure for applying the algorithm to tune the controller gains.

Step 1: Initialization

The authors specify the value ranges for the PID parameters based on engineering experience and set the initial values for the algorithm parameters, such as population size, fluorescence brightness, and attraction.

Areas requiring further clarification:

1. Justification for parameter ranges and initial values: The authors should discuss the rationale behind the chosen parameter ranges and initial values, and consider conducting sensitivity analyses to assess their impact on the optimization results.

Answer 1. Thank you for your valuable comments. After consulting the references and discussing with the authors, we have summarized the following replies:

Proportional gain ($k_{p}$): The proportional gain determines the direct response strength of the controller to the error. A larger $k_{p}$ can make the controller more sensitive to error changes, thereby responding to error changes faster. In some applications, a higher $k_{p}$ value may be required to achieve fast response and lower steady-state error. Therefore, setting the range of $k_{p}$ to [0, 100] can ensure that the controller can find a suitable proportional gain under different operating conditions.

Integral gain ($k_{i}$): The integral gain is used to eliminate steady-state errors, but a larger integral gain may cause system instability or oscillation. Therefore, the range of $k_{i}$ is usually set smaller to ensure that the integral action does not excessively affect the stability of the system.

Derivative gain ($k_{d}$): The derivative gain is used to predict the trend of error changes and provide a pre-correction effect. A larger $k_{d}$ may lead to excessive amplification of noise, thereby affecting the stability of the system. Therefore, $k_{d}$ is usually set smaller. Setting the range of $k_{d}$ to [0, 1] can effectively limit the differential action and prevent the system from being too sensitive to noise.

The PID controller parameter $k_{p}\in[0,100],k_{i}\in[0,1],k_{d}\in[0,1]$ is set to improve the efficiency and feasibility of the optimization algorithm while ensuring the performance and stability of the control system.

We used MATLAB to perform a sensitivity analysis and came to the following conclusions:

The range selection of all three parameters takes into account the stability of the system. A larger $k_{p}$ range provides flexibility, but requires careful adjustment to prevent instability. Smaller $k_{i}$ and $k_{d}$ ranges help control the strength of the integral and differential actions, avoiding system oscillation and oversensitivity to noise. A larger $k_{p}$ range allows the system to respond quickly, and smaller $k_{i}$ and $k_{d}$ ranges ensure that the system can eliminate steady-state errors and improve dynamic performance without over-response and oscillation. Reasonable parameter range settings can reduce the search space of the optimization algorithm and improve optimization efficiency. By limiting the range of $k_{i}$ and $k_{d}$, suitable parameter combinations can be found more quickly without wasting computing resources in invalid parameter spaces.

Step 2: Iteration

The authors describe the iterative process of updating the particle positions based on the attraction and density calculations introduced in the previous sections, which is consistent with the general structure of the firefly algorithm.

Areas requiring further clarification:

1. Termination criteria: The authors should provide clear guidelines on when to stop the optimization process, such as specifying the maximum number of iterations or the convergence threshold.

Answer 1. We used the MATLAB program to determine the maximum number of iterations and the convergence threshold. Define the maximum number of iterations. According to the complexity of the problem and the limitation of computing resources, a reasonable maximum number of iterations is selected. The maximum number of iterations is defined as a variable so that it can be referenced and checked during the optimization process. Then the algorithm is initialized. During the optimization process, the population solution needs to be initialized first. Each solution represents a combination of PID parameters. An evaluation function is defined to evaluate the quality of each solution and select an appropriate initial solution. Finally, the optimization process is performed to find the maximum number of iterations. The convergence threshold is also tested many times until the most suitable result is found.

Step 3: Termination

The optimization process terminates when either the maximum number of iterations is reached or the ratio of high-likelihood particles exceeds a predefined threshold.

Areas requiring further clarification:

1. Trade-off between termination criteria: The authors should analyze the impact of the maximum number of iterations and the high-likelihood particle ratio threshold on the optimization results and propose strategies for balancing the exploration and exploitation aspects of the algorithm.

Answer 1. Thank you for your valuable comments on this part. Due to the length of the article, we only made a brief introduction in the article. We will elaborate on the impact of the two on the optimization results in the following reply:

If the maximum number of iterations is set too small, the algorithm may stop before fully exploring the solution space, resulting in the solution found may not be the global optimal solution. Increasing the number of iterations can usually increase the possibility of finding a better solution, because the algorithm has more opportunities to explore the solution space and optimize. However, too many iterations may also lead to overfitting or waste of computing resources. Fewer iterations can reduce computing time and resource consumption, which is suitable for situations where approximate solutions need to be obtained quickly. More iterations will increase computing time and resource consumption, which is suitable for situations where the quality of the solution is required to be high.

The high likelihood particle ratio threshold refers to the threshold used to determine which particles are considered to have high probability during the optimization process, which usually determines what proportion of particles will be retained or updated. A lower threshold will retain more particles, which can increase the diversity and exploration of the search and help avoid local optimal solutions. However, retaining too many particles may cause the search process to be too dispersed and difficult to concentrate on finding the best solution. A higher threshold will retain fewer high-probability particles, which may make the search process more concentrated and help accelerate convergence. However, too high a threshold may lead to insufficient particle diversity and increase the risk of falling into a local optimal solution.

By reasonably setting the maximum number of iterations and the high likelihood particle ratio threshold, the performance of the elastic firefly algorithm in PID parameter optimization can be effectively improved, which not only ensures the optimization quality but also controls the consumption of computing resources.

Optimization Results:

The elastic firefly algorithm reached the termination condition after 67 iterations, resulting in the optimized PID parameters: kp = 47.832, ki = 0.718, and kd = 0.697.

Areas requiring further analysis and discussion:

1. Comparison with other methods: The authors should benchmark their approach against existing methods to highlight its advantages and limitations.

Answer 1. Thank you for your valuable suggestions. We, the authors, have supplemented and improved the information comparing the elastic firefly algorithm with existing methods (taking genetic algorithm and particle swarm optimization algorithm as examples), and explained their advantages and limitations, which will enable readers to have a better understanding of this article.

2. Robustness to variations: The authors should analyze the sensitivity of the controller's performance to parameter uncertainties and propose strategies for ensuring robust and stable operation.

Answer 2. Thank you for your valuable comments. Due to the length of the article, only a brief discussion is made in the article. The detailed discussion is introduced as follows: Apply small disturbances to the key parameters of the controller (such as gain, time constant, phase compensation, etc.) and observe the changes in system performance. Use simulation tools (such as MATLAB/Simulink) to simulate the control system multiple times and record the changes in performance indicators (such as overshoot, steady-state error, response time, etc.). Draw the Nyquist diagram and Bode diagram of the system and analyze the gain margin and phase margin of the control system. Systems with smaller gain margin and phase margin are more sensitive to parameter uncertainty. Calculate the sensitivity function and compensation sensitivity function and analyze their amplitudes at different frequencies. Higher sensitivity means that the system is more sensitive to parameter changes.

Strategies to ensure robust and stable operation: (1) Adaptive control: Design an adaptive controller that can adjust parameters based on real-time data to adapt to dynamic changes in the system. Use real-time parameter estimation and self-adjustment mechanisms to enable controller parameters to adjust as the system changes. (2) Parameter optimization: Use genetic algorithms to optimize controller parameters and find the optimal parameter combination to improve system performance and robustness. (3) Increase redundancy and fault-tolerant design: Design a redundant control system so that when one controller fails, the other controller can continue to work. Integrate fault detection and isolation mechanisms to promptly detect and isolate faults in the control system to ensure stable system operation.

3. Computational complexity and convergence properties: The authors should discuss the scalability of their approach and its suitability for real-time implementation on resource-constrained platforms.

Answer 3. Thank you for your valuable comments. Due to the length of the article, only a brief discussion is made in the article. The detailed discussion is introduced as follows: The elastic firefly algorithm performs well in solving various complex optimization problems, but its real-time implementation on resource-constrained platforms may face some challenges. I think scalability can be considered from the aspects of algorithm complexity, parallelization, and adaptability to different problem sizes. Algorithm complexity is mainly divided into time complexity and space complexity. The computing tasks can be assigned to multiple computing nodes. Each node independently performs part of the computing tasks, exchanges information through network communication, and finally summarizes the results. Distributed computing can effectively handle large-scale problems. In terms of space complexity, the search space is divided into several subspaces, and independent firefly algorithm calculations are performed in each subspace, and the results are finally merged. This can reduce the number of fireflies in each subspace and reduce the computational complexity. The computational process of the elastic firefly algorithm is highly parallel, because the movement and brightness update of each firefly can be calculated independently. The use of parallel computing technology (such as GPU acceleration and distributed computing) can significantly improve the scalability of the algorithm. Adapting to different problem scales is an advantage of the elastic firefly algorithm. By adjusting parameters such as the number of fireflies, the number of iterations, and the step size factor, it can flexibly respond to problems of different scales and complexities and improve the adaptability of the algorithm. For complex multi-level problems, a hierarchical optimization strategy can be adopted, first performing rough optimization at the macro level, and then fine optimization at the micro level to improve the efficiency of the algorithm.

On resource-constrained platforms, such as embedded systems and mobile devices, computing resources are limited. The implementation of the optimization algorithm requires efficient use of the CPU and GPU to minimize the computational burden. The low-power algorithm design is used to reduce unnecessary power consumption during the calculation process and improve the battery life of the system.

REVIEW OF SECTION 4.1:

The authors present the establishment of a simulation model for the mining concrete mixing and transport tank truck using a state-space representation and a schematic diagram. They also provide a detailed list of system parameters that characterize the vehicle's physical properties.

State-Space Model:

Equation (47) introduces a state-space model of the system, where the state variable x includes the vehicle speed ξ, acceleration dξ/dt, and other related variables. The model is expressed as a matrix differential equation, with coefficient matrices M, C, and N, and an additional term w representing the relative motion of each order of mass in the equivalent system.

Areas requiring further clarification:

1. Derivation of coefficient matrices: The authors should discuss the process of system identification and the assumptions made in obtaining the coefficient matrices M, C, and N from the physical properties of the mining concrete mixing and transport tank truck.

Answer 1. Thank you for your valuable suggestions. We have supplemented and improved the system identification process in this part of the article. Due to the length and conciseness of the article, we cited two references in this part to give a detailed description of the sources of the coefficient matrices $M$, $C$, and $N$ of the matrix differential equation.

Simulation Model Schematic:

Figure 5 presents a schematic diagram of the dynamic simulation model, including an equivalent mechanical model of liquid sloshing, a dynamic model of the concrete mixing transport tanker, and a display of the vehicle's lateral acceleration.

Areas requiring further clarification:

1. Definition of variables: The authors should provide a clear definition of the variables U, θ, and ω in the schematic and explain their role in the simulation model.

Answer 1. Thank you for your valuable comments on this part of the article. We have improved the annotations of all variables $\frac{\text{d}U}{\text{d}t}$, $h$, $\delta$, $u$, $\theta$ and $\omega_{1}$ in the dynamic simulation model of Figure 5. This will give readers a clearer understanding of this part. Due to the length of the article, we will explain their role in the simulation model below:

(1) Lateral acceleration $\frac{\text{d}U}{\text{d}t}$ of the semitrailer: The lateral acceleration is a key parameter of the vehicle when turning and moving laterally, which directly affects the lateral stability of the vehicle. Large lateral acceleration may cause the vehicle to roll over or skid, especially when the load is heavy. By analyzing the lateral acceleration, the stability of the vehicle at different turning radii and speeds can be predicted and evaluated. The suspension system of the vehicle can be designed and optimized to improve the stability of the vehicle under different working conditions.

(2) Liquid depth $h$: The liquid depth (i.e., the liquid level of the concrete in the tank) has an important influence on the dynamic behavior and performance of the vehicle. Changes in the liquid depth will affect the center of gravity of the concrete, which in turn affects the longitudinal and lateral stability of the vehicle. A higher liquid depth may cause the center of gravity of the vehicle to be higher, increasing the risk of rollover. When turning, changes in liquid depth will change the flow characteristics of concrete, thereby affecting the lateral stability of the vehicle. Excessive liquid depth may cause increased concrete shaking and reduce lateral stability.

(3) Tractor front wheel steering angle $\delta$: In the dynamic simulation model of mining concrete mixer transport tank trucks, the tractor front wheel steering angle (commonly referred to as steering angle) plays an important role in the vehicle's handling, stability and overall performance. The steering angle of the front wheel determines the vehicle's turning radius and affects the vehicle's ability to drive under different curve radii. A larger steering angle allows the vehicle to drive within a smaller turning radius, improving the vehicle's flexibility. Steering response: The steering angle affects the vehicle's response to the driver's control. A suitable steering angle can improve the vehicle's response speed and stability when turning.

(4) Vehicle speed $u$: Vehicle speed plays an important role in the vehicle's dynamic behavior and overall performance. Higher vehicle speeds usually increase the vehicle's turning radius, making it more difficult for the vehicle to turn at high speeds. The effect of vehicle speed on the turning radius may also cause oversteering or understeering, affecting handling. Changes in vehicle speed will affect the response characteristics of the front wheel steering. At high speeds, the steering response becomes more sensitive, while at low speeds it is relatively slow.

(5) Articulation angle $\theta$: The articulation angle (also known as the articulation angle between the tractor and the semitrailer) has a significant impact on the vehicle's handling, stability and overall performance. The articulation angle determines the semitrailer's turning radius. A larger articulation angle generally increases the turning radius, requiring more space for the vehicle to turn. Conversely, a smaller articulation angle reduces the turning radius and improves turning flexibility. The articulation angle affects the vehicle's handling response. Proper articulation angle settings can improve the vehicle's handling stability, especially on complex roads and narrow spaces.

(6) Tractor yaw rate $\omega_{1}$: The yaw rate (also known as the yaw rate) of the tractor plays an important role in the vehicle's handling, stability and overall performance. The yaw rate is the speed at which the vehicle rotates when turning or changing lanes. A higher yaw rate may cause the vehicle's lateral force to increase, thereby affecting the vehicle's stability. When driving at high speeds or making sharp turns, excessive yaw rates may cause the vehicle to lose control. The yaw rate affects the side slip of the vehicle. The side slip angle of the vehicle (i.e. the angle between the tire and the direction of travel) is directly related to the yaw rate. If the yaw rate is too large, the vehicle may slip, affecting driving safety.

2. Integration of liquid sloshing and vehicle dynamics: The authors should clarify how the equivalent mechanical model of liquid sloshing and the dynamic model of the concrete mixing transport tanker are integrated and how the liquid sloshing dynamics influence the overall vehicle dynamics.

Answer 2. Integration of liquid sloshing and vehicle dynamics: The author should clarify how to integrate the equivalent mechanical model of liquid sloshing and the dynamic model of concrete mixer truck, and how the liquid sloshing dynamics affects the overall vehicle dynamics.

Thank you for your valuable suggestions. We will supplement and improve according to your suggestions. However, due to the length and conciseness of the article, we will explain and illustrate in detail as follows:

Steps to integrate the equivalent mechanical model of liquid sloshing and the vehicle dynamic model:

(1) Input of liquid mechanical model:

The mass distribution, center of gravity position, and forces caused by the liquid (such as lateral and longitudinal forces) are used as inputs and integrated into the dynamic model of the vehicle.

(2) Dynamic influence of liquid:

The influence of liquid sloshing and flow on the vehicle (such as the dynamic center of gravity change caused by the liquid, the effect of the liquid on the lateral and longitudinal forces of the vehicle) is integrated into the vehicle's motion equations.

(3) Coupled dynamic analysis:

Consider the additional dynamic effects caused by liquid sloshing, such as how the inertial force of the liquid affects the stability and handling of the vehicle when turning or accelerating sharply.

Use simulation tools (such as MATLAB/Simulink) to combine the liquid model with the vehicle model for comprehensive dynamic analysis.

(4) Adjust model parameters:

Adjust the parameters of the liquid model and vehicle model according to the actual situation to ensure that the simulation results are consistent with the actual situation. For example, adjust according to factors such as liquid volume, tank shape, and vehicle speed.

The sloshing of the liquid will cause lateral forces to be generated on the inner wall of the tank, which will affect the lateral stability of the vehicle. When making sharp turns or driving at high speeds, the inertia of the liquid may cause the vehicle to lose control or skid. The flow of the liquid will cause the center of gravity of the vehicle to change over time, thereby affecting the stability of the vehicle. The dynamic center of gravity change of the liquid may cause the vehicle body to tilt or sway, affecting the handling and comfort. During acceleration or braking, the inertial force of the liquid will affect the longitudinal stability of the vehicle. For example, the forward and backward movement of the liquid may cause the braking distance to change or the acceleration performance to change. The sloshing of the liquid may cause the vehicle body to sway laterally and longitudinally, affecting the comfort and handling of the vehicle. Especially on uneven roads, the swaying of the liquid may cause the vibration of the vehicle to increase.

3. Computational tools and considerations: The authors should provide information on the software or programming environment used to implement the simulation model and discuss any specific considerations for numerical integration or solver settings.

Answer 3. Thank you for your valuable suggestions. Adding simulation programming environment information can make our article more complete. We have added this information in this part of the article: "This study uses R2023a Win64 version of MATLAB software, the operating environment is Windows 11 system, and the processor is Intel(R) Core(TM) i7-14700HX 2.10 GHz." And specifically describe the role of the tools: Simscape and Vehicle Dynamics Toolbox, and simulate fluid dynamics and vehicle model establishment and simulation respectively.

Further discuss any specific considerations for numerical integration or solver settings. Numerical integration methods are divided into explicit integration methods, implicit integration methods, and adaptive step methods. Explicit integration methods have the advantages of simple implementation and are suitable for non-rigid systems, but they are unstable for rigid systems and may require a very small step size to ensure stability. Implicit integration methods are more stable for rigid problems and are suitable for dealing with rapidly changing systems, but the computational complexity is high and may require solving nonlinear equations. The adaptive step method automatically adjusts the step size according to the dynamic behavior of the system to improve computational efficiency and accuracy, but it may lead to increased computational overhead, especially when dynamic changes are drastic. The setting of the solver is usually accompanied by indicators such as stiffness, error control, step size selection, stability and convergence. For non-stiff systems, explicit solvers can be used, such as ode45 in MATLAB. Explicit solvers are suitable for dealing with rapidly changing systems and have high computational efficiency, but require a smaller time step to ensure stability. For stiff systems, implicit solvers are more suitable, such as ode15s in MATLAB. Implicit solvers are more stable when dealing with stiff problems and can use larger time steps, but the computational complexity is higher. Error control is divided into relative error and absolute error. The relative error tolerance controls the relative error of the solver at each time step. A lower relative error tolerance can improve the calculation accuracy, but may increase the calculation time. The absolute error tolerance controls the absolute error of the solver at each time step. When the variable value is small, a lower absolute error tolerance helps maintain accuracy. The adaptive step size algorithm can automatically adjust the time step according to the dynamic characteristics of the system, thereby improving computational efficiency and accuracy. In the case of large dynamic changes, the adaptive step size algorithm can shorten the time step to ensure accuracy, and when the system changes slowly, the time step can be increased to improve efficiency.

System Parameters:

Table 1 lists the system parameters of the mining concrete mixing and transport tank truck, including its dimensions, weight, engine specifications, and other relevant properties.

Areas requiring further discussion:

1. Source and methodology: The authors should clarify the source or methodology used to obtain the listed parameters, whether they are based on manufacturer specifications, experimental measurements, or estimates from similar vehicles.

Answer 1. Thank you for your valuable suggestions. We have clearly marked the sources of the listed parameters and marked them as coming from the Internet. This will allow readers to have a clearer understanding of this part.

2. Sensitivity analysis: The authors should analyze the sensitivity of the model's predictions to changes in key parameters and propose strategies for robust simulation and design.

Answer 2. Thank you for your valuable suggestions. We will make modifications according to your requirements. Due to the length and simplicity of the article, we will give a detailed answer to this question as follows: Although the parameters of the KJCJ-4 mining concrete mixer transport tank truck contain many parameters, the purpose of listing its parameters is not only to discuss the changes in the parameters affected by this model, but also to enable readers to have a more intuitive and in-depth understanding of this vehicle. This simulation model has a certain impact on some parameters of the KJCJ-4 mining concrete mixer transport tank truck, which are described in detail in the following simulation diagrams. The main impacts are vehicle mass, liquid depth, vehicle speed, steering angle and articulation angle. Sensitivity analysis is to observe the changes in system output by changing each key parameter one by one, keeping other parameters unchanged. I mainly want to explain the strategy of robust simulation and design, introduce parameter uncertainty in the simulation model, and evaluate the robustness of the system to uncertainty through methods such as Monte Carlo simulation. Consider the range of parameter changes during design to ensure that the system can still work normally within these ranges. These strategies include improving model robustness, improving control strategies and optimizing design parameters to ensure the stability and performance of the system under different operating conditions.

3. Validation and verification: The authors should compare the simulated vehicle's performance with experimental measurements or field observations to assess the accuracy and reliability of the simulation model.

Answer 3. Thank you for your valuable comments on this section. We will modify and improve this section based on your comments, add more details on comparing the performance of the simulated vehicle with experimental measurements or field observations, and also explain the comparison between simulation and actual experiments in the final conclusion and outlook, which further highlights the accuracy of the model.

Comparing the performance of the simulated vehicle with experimental measurements or field observations is a key step in verifying the accuracy and reliability of the simulation model. By collecting experimental data, performing simulations, comparing results, analyzing deviations, adjusting models, and evaluating reliability, it is possible to ensure that the simulation model accurately reflects the actual performance of the vehicle, thereby providing valuable support in the design and optimization process.

REVIEW OF SECTION 4.2:

The authors present a control simulation system experiment for the mining concrete mixing tank truck using MATLAB/Simulink. They develop a multi-factor simulation model that integrates the tank truck dynamics, hydrostatic drive system, rock slope model, and the elastic FA algorithm optimized filter adaptive PID controller. The authors conduct simulation experiments to compare the performance of the proposed controller with the traditional PID controller under various operating conditions.

Simulation Model Development:

The development of a comprehensive simulation model that considers multiple factors affecting the mining concrete mixing tank truck's performance is a significant contribution of this study. The model incorporates the vehicle dynamics, hydrostatic drive system, rock slope model, and the proposed adaptive PID controller optimized by the elastic FA algorithm.

Areas requiring further clarification:

1. Detailed description of model architecture: The authors should provide a more detailed description of the model architecture and the key Simulink blocks employed to enhance the reproducibility of their work.

Answer 1. Thank you for the valuable suggestions from the experts. We apologize that we mentioned the key Simulink blocks but did not give a detailed description of them. We supplemented and improved the detailed description of the key Simulink blocks and explained the subsystems of the entire model architecture. "Simulink is a graphical programming environment provided by MATLAB for modeling, simulating, and analyzing dynamic systems. It uses graphical block diagrams to build and connect system models, and each block represents a function or operation. The entire model architecture is divided into dynamics subsystem, liquid sloshing subsystem, controller subsystem, environment subsystem, data acquisition and recording subsystem, etc." Doing this can make this paragraph more complete.

Step Response Experiment (Figure 7):

The results demonstrate that the proposed controller achieves a faster settling time (5.4s) and a lower steady-state error (0.01km/h) compared to the traditional PID controller, while also eliminating overshoot.

Areas requiring further discussion:

1. Selection of step signal amplitude: The authors should provide more information on the selection of the step signal amplitude and the rationale behind it.

Answer 1. Thank you for your valuable comments. Step signals are often used in time domain analysis, and the transient response characteristics of the system can be clearly observed, including rise time, peak time, overshoot and steady-state error. This helps in debugging and optimizing PID controller parameters. From a safety perspective, mining concrete mixing tank trucks usually operate in complex and dangerous mining environments. Lower speeds ensure greater safety and avoid vehicle loss of control and accidents. The low speed can make the operation more stable, especially in situations where precise positioning and operation are required, such as driving in narrow mine tunnels or unloading materials at designated locations. Lower speed can reduce the stress on the vehicle's mechanical components, extend the service life of the vehicle, and reduce maintenance costs. The PID controller can control the speed more accurately, reduce overshoot and steady-state errors, and ensure the smoothness of the system response. The step signal value range of 0~5km/h is selected because the mining concrete mixing tank truck has a minimum speed that can change significantly when the speed reaches 5km/h. An excessive speed range will not only cause Experiments are helpful, but they also cause waste. If the speed range is too small, there will be no obvious effect. This speed value range is the best speed value range we have obtained after many simulation experiments. Therefore, this value selection is more in line with actual open pit mine production.

2. Implications of improved performance: The authors should discuss the implications of the improved settling time and steady-state error on the overall performance and efficiency of the mining concrete mixing tank truck.

Answer 2. Impact of performance improvements: The authors should discuss the impact of improved settling time and steady-state error on the overall performance and efficiency of mining concrete mixer tank trucks.

Thank you for your valuable opinions. We will answer your questions based on your questions. Due to the length of the article, we will describe it in detail as follows: Steady-state error refers to the difference between the actual output and the expected target after the system reaches steady state. gap. For the speed control system of mining concrete mixing tank trucks, the steady-state error has a significant impact on the overall performance and efficiency. Steady-state error will affect the control accuracy of the system. For concrete mixing tank trucks, precise speed control is crucial to maintain mixing uniformity and avoid solidification of concrete. Large steady-state errors may lead to uneven mixing of concrete or unstable transportation speed.

Ramp Driving Experiment (Figure 8):

The results show that the proposed controller maintains a smaller speed tracking error (0.04 km/h) compared to the traditional PID controller (0.22 km/h) when entering and exiting the ramp, demonstrating faster convergence and better disturbance rejection capabilities.

Areas requiring further clarification:

1. Ramp profile and duration: The authors should provide more details on the ramp profile and the duration of the ramp driving experiment.

Answer 1.Thank you for your valuable comments on this section. We will modify and improve this section based on your comments. The slope is the inclination angle of the slope, usually expressed in percentage or degree. It reflects the steepness of the open-pit mine ramp. By designing the slope profile and the test duration in detail, the speed tracking performance of the mining concrete mixer transport tank truck on the slope can be fully evaluated. This information is of great significance for optimizing the vehicle control system and improving safety and productivity. Through the analysis of experimental data, the control algorithm can be further optimized to ensure that the vehicle can operate safely and efficiently under different slope conditions.

2. Impact on safety and productivity: The authors should discuss the impact of the improved speed tracking performance on the safety and productivity of the mining operation, considering the challenges posed by steep slopes and uneven terrain.

Answer 2. Thank you for your valuable comments on this section. We will modify and improve this section based on your comments, supplement the role and influence of speed tracking performance on the safety and production efficiency of open-pit mine production operations, and provide additional references to support the article, making it very persuasive. "The speed tracking performance of mining concrete mixing and transport tank trucks [46] has an important impact on the safety and production efficiency of open-pit mine production operations. Accurate speed tracking performance ensures that the vehicle can travel at a predetermined speed and avoids sudden acceleration or deceleration, thereby improving the stability of operation. In the complex and dangerous environment of the mining area, accurate control of vehicle speed can reduce the risk of collision and rollover. By optimizing the control algorithm, regular maintenance and training operators, the speed tracking performance can be effectively improved, thereby improving the safety and production efficiency of operations. In actual operation, stable speed control can not only reduce the risk of accidents, but also improve transportation efficiency and concrete quality, ensuring the smooth progress of open-pit mine production operations."

Comprehensive Driving Experiment (Figure 9):

The results demonstrate that the proposed controller maintains a lower average speed control error (0.03 km/h) and maximum speed error (0.17 km/h) compared to the traditional PID controller, while also exhibiting better disturbance rejection and adaptability to varying road conditions.

Areas requiring further discussion:

1. Characteristics of white noise interference: The authors should provide more information on the characteristics of the white noise interference and its relevance to real-world driving conditions.

Answer 1. Thank you for your valuable comments on this section. We will modify and improve this section based on your comments, supplement the characteristics of white noise interference and its correlation with actual driving conditions, and verify it through additional references. White noise refers to noise whose power spectrum density is constant in the entire frequency domain. Random noise with the same energy density at all frequencies is called white noise. White noise has the characteristics of uniform spectral distribution and randomness, and has a wide range of effects on the control system, vibration and noise, concrete mixing quality, equipment failure and maintenance, and control and driving of mining concrete mixing tank trucks. By enhancing the robustness of the control system, improving the vibration and sound insulation of the vehicle, regular maintenance inspections, and training operators, white noise interference can be effectively dealt with and the performance and stability of the vehicle can be improved. This improvement will enable readers to understand what white noise is and have a clearer understanding of this article.

2. Implications of improved performance: The authors should discuss the implications of the improved speed control accuracy and robustness on the overall efficiency and reliability of the mining concrete mixing tank truck.

Answer 2. Thank you for your valuable comments on this section. We will modify and improve this section based on your opinions, and add a supplementary explanation of the impact of improved speed control accuracy and robustness on the overall efficiency and reliability of mining concrete mixing tank trucks. Elastic FA optimization filter adaptive PID control can significantly improve the speed control accuracy and robustness of mining concrete mixing tank trucks. This control strategy not only improves the control accuracy of the concrete mixing tank truck under various operating conditions, but also enhances the robustness of the system, allowing it to operate stably in complex environments. Through these improvements, the overall efficiency and reliability of concrete mixer tank trucks have been significantly improved, which is reflected in increased production efficiency, reduced failure rates, reduced maintenance costs and extended equipment service life. These benefits are of great significance to improving the overall operating efficiency of mining concrete mixing tank trucks and reducing operating costs. This improvement will enable readers to understand the impact of improved speed control accuracy and robustness on the overall efficiency and reliability of mining concrete mixing tank trucks, and have a clearer understanding of this article.

Dynamic Disturbance Experiment (Figure 10):

The results show that the proposed controller maintains a smaller speed tracking error (within 0.3 km/h) compared to the traditional PID controller (up to 0.47 km/h) when subjected to fluid sloshing disturbances, demonstrating better adaptability and disturbance rejection capabilities.

Areas requiring further clarification:

1. Modeling of fluid sloshing disturbance: The authors should provide more details on the characteristics of the fluid sloshing disturbance and how it is modeled in the simulation.

Answer 1. Thank you for your valuable comments on this section. We will modify and improve this section based on your comments, and supplement the details about the fluid sloshing disturbance characteristics and how they are modeled in the simulation. The article describes the fluid sloshing disturbance characteristics of mining concrete mixing tank trucks and their modeling, which involves aspects such as fluid dynamics, vehicle dynamics, and control system modeling. The model can accurately describe the behavior of fluid sloshing, which has an important impact on the stability of the tank truck, the quality of concrete, and the design and performance of the control system. By using physical modeling, numerical simulation, and control system modeling, we can better cope with the challenges brought by fluid sloshing and improve the overall efficiency and reliability of mining concrete mixing tank trucks. This improvement will enable readers to understand the details of the fluid sloshing disturbance characteristics and how they are modeled in the simulation, and have a clearer understanding of this article.

2. Impact on stability and safety: The authors should discuss the potential impact of fluid sloshing on the stability and safety of the mining concrete mixing tank truck, and highlight the significance of the improved speed tracking performance in mitigating these risks.

Answer 2. Thank you for your valuable comments on this section. We will modify and improve this section based on your opinions. We will also add the potential impact of fluid sloshing on the stability and safety of mining concrete mixing tank trucks and the impact of improving speed tracking performance on mitigating the significance of these risks. Fluid sloshing has a significant impact on the stability and safety of mining concrete mixing tank trucks, which may cause the vehicle to roll, increase control difficulty, reduce concrete quality, and increase the risk of accidents. Improving speed tracking performance can effectively mitigate these risks, ensure stable vehicle travel, reduce fluid turbulence, optimize the concrete mixing process, and enhance overall safety. By improving the control system and implementing real-time adjustments and feedback mechanisms, the accuracy and robustness of speed control can be significantly improved, thereby improving the stability and safety of mining concrete mixer trucks. This improvement will give readers a clearer understanding of this article.

Criticism and Suggestions:

1. Detailed information on simulation model implementation: The authors should provide more detailed information on the implementation of the simulation model in MATLAB/Simulink, including the specific blocks and libraries used for each component, to enhance the reproducibility and transparency of their work.

Answer 1. Thank you for your valuable comments on this part. We will modify and improve this part based on your comments. We will add more detailed information about implementing the simulation model in MATLAB/Simulink and the impact of fluid sloshing on mining concrete mixer tank trucks. It has a significant impact on the stability and safety of the vehicle, which may lead to vehicle roll, increased difficulty in handling, reduced concrete quality and increased risk of accidents. Improving speed tracking performance can effectively mitigate these risks, ensure stable vehicle travel, reduce fluid turbulence, optimize the concrete mixing process, and enhance overall safety. By improving the control system and implementing real-time adjustments and feedback mechanisms, the accuracy and robustness of speed control can be significantly improved, thereby improving the stability and safety of mining concrete mixer trucks. This improvement will give readers a clearer understanding of this article.

2. Discussion of simulation parameters: The authors should discuss the selection of the simulation parameters, such as the step signal amplitude, ramp profile, and white noise characteristics, and justify their relevance to real-world operating conditions.

Answer 2. Thank you for your valuable comments on this section. We will modify and improve this section based on your comments. Due to the length of the article, we will explain and interpret it in detail as follows.

Step signals are used to simulate the system's response to sudden input changes, such as a sudden increase in load or an instantaneous change in the control signal. In vehicle dynamics simulation, step signals can be used to test the system's ability to respond to sudden changes, such as acceleration or braking operations. The amplitude of the step signal should be selected to match the actual operating conditions. For mining concrete mixer tank trucks, the amplitude of the step signal should simulate the acceleration or deceleration amplitude in actual operation. Selecting too large an amplitude may result in unrealistic system response, while selecting too small an amplitude may not effectively test the dynamic performance of the system.

Ramp profiles are used to simulate the system's response to gradually changing input conditions, such as slow acceleration or deceleration. In vehicle simulation, ramp profiles can simulate the gradual acceleration or deceleration of tank trucks during actual driving. The ramp profile can be set based on the acceleration and deceleration data in actual operation. For example, if the actual acceleration process takes 15 seconds to accelerate from 0 to 5 km/h, then set the time constant of the ramp profile to 15 seconds in the simulation to ensure that the simulation results are consistent with the actual operation.

White noise is a random signal containing all frequency components, which is used to simulate random interference or measurement noise in the system. In vehicle simulation, white noise can be used to test the robustness of the system to random interference and evaluate the stability of the system in the actual environment. The characteristics of white noise can be determined by analyzing the noise data or interference level in the actual environment. For example, if the noise amplitude in the actual environment is between 0.1 and 0.5, the white noise amplitude in the simulation should be set in this range to ensure the practical relevance of the simulation results.

The selection of step signal, ramp profile, and white noise should be based on the actual operating conditions to ensure that the simulation results can reflect the system behavior in the real environment. Verify whether the selection of simulation parameters is reasonable by comparing with the actual operating data. When implementing simulation models in MATLAB/Simulink, the reasonable selection of step signal amplitude, ramp profile, and white noise characteristics is crucial to ensure the practical relevance of simulation results. By setting parameters that match actual operating conditions and conducting sufficient testing and verification, we can ensure that the simulation model accurately reflects the behavior of the system in the real environment. This not only improves the realism of the simulation, but also enhances the reliability of the model.

3. Comprehensive analysis of implications: The authors should provide a more comprehensive analysis of the implications of the improved control performance on the safety, productivity, and efficiency of the mining operation, relating the simulation results to the specific challenges and requirements of mining concrete mixing tank trucks.

Answer 3. Thank you for your valuable opinions on this section. We will modify and improve this section based on your opinions. Due to the length of the article, we will elaborate and interpret it in detail as follows.

The elastic firefly algorithm optimized filtering adaptive PID control has a significant impact on the safety, productivity and efficiency of concrete mixing tank trucks used in open pit mines. The adaptive PID controller optimized by the elastic firefly algorithm can more accurately adjust the speed and direction of the mixing tank truck, thereby reducing operating errors and reducing safety hazards caused by unstable speed or slow response. By optimizing the filter, the adaptive PID controller can handle noise and interference more effectively, reduce the failure rate of equipment in harsh environments, and improve overall safety. The optimized PID controller can respond to operating instructions more quickly and accurately, reducing the acceleration and deceleration time of the mixing tank truck, thereby improving the efficiency of concrete transportation and mixing. More precise control can optimize the working status of the engine and drive system and reduce energy waste, thereby reducing fuel consumption and operating costs. The optimized control system can maintain the smooth operation of tank trucks and reduce the waste of resources and decrease in production efficiency caused by unstable operation.

The operating environment of open-pit mines is complex and harsh, such as uneven terrain, large climate changes, flying dust, etc., which places high requirements on the stability and control accuracy of concrete mixing tank trucks. In the operation of mining concrete mixer tank trucks, the load changes frequently, such as when loading and unloading concrete. Drastic changes in load will affect the control performance of the vehicle. By improving the control strategy, it is possible to improve operational accuracy, ensure the quality and consistency of concrete during transportation, and meet the high standards of construction. After further simulation, the optimized controller can respond faster to input changes and load fluctuations, improving the dynamic performance and operating efficiency of the system. The improved control system is more robust to environmental disturbances and load changes, reducing system failure rates and maintenance requirements.

By improving control accuracy, responsiveness and robustness, the specific challenges of open pit mines can be effectively addressed and the operational stability and efficiency of tank trucks can be improved. The simulation results verify the relevance and effectiveness of these optimization measures in actual operations, and provide theoretical support and practical basis for improving the overall performance of mining concrete mixing tank trucks.

4. Sensitivity analyses: The authors should consider conducting sensitivity analyses to evaluate the robustness of the proposed controller to variations in system parameters, such as vehicle mass, tire properties, and fluid characteristics, to provide insights into the controller's adaptability to different operating scenarios.

Answer 4. Thank you for your valuable comments on this section. We will modify and improve this section based on your comments. Due to the length of the article, we will elaborate and interpret it in detail as follows.

Establish a simulation model: Establish a dynamic model of a mining concrete mixer truck in MATLAB/Simulink, including vehicle dynamics, tire characteristics, and fluid dynamics. Implement a filter adaptive PID controller optimized by the elastic firefly algorithm and integrate it into the model.

Select system parameters: vehicle mass, tire characteristics, fluid characteristics

Design test scenarios: Set a benchmark test scenario under normal operating conditions. Design different test scenarios, simulate by changing the values of the above parameters, and observe the performance of the controller.

Perform simulation: Run the simulation under different parameter settings and record the key performance indicators of the control system, such as stability, response time, overshoot, and tracking accuracy. Collect performance data under different test scenarios for comparative analysis.

Compare the accuracy and stability of the control system under different parameter settings to analyze whether the controller can maintain high-precision control performance. Evaluate the response time of the controller under different parameter conditions to ensure fast response under various operating conditions. Evaluate the robustness of the controller by comparing the stability under various parameter settings. For example, analyze whether the control system remains stable under load changes, tire friction coefficient changes, and fluid characteristics changes. Evaluate the adaptability of the controller in different operating scenarios to ensure its reliability and effectiveness in actual mining environments.

Based on the results of the sensitivity analysis, make improvement suggestions, such as adjusting the parameter range of the PID controller, improving system modeling, or introducing new control strategies. Based on the analysis results, adjust the parameter settings of the actual system to ensure the performance optimization of the controller under different operating conditions.

This analysis can provide an in-depth understanding of the robustness of the filter adaptive PID controller optimized by the elastic firefly algorithm to vehicle mass, tire characteristics, and fluid characteristics, and ensure its effectiveness in different operating scenarios. This will help improve the overall performance and reliability of mining concrete mixer tank trucks.

5. Comparison with state-of-the-art control techniques: The authors should compare the performance of the proposed controller with other state-of-the-art control techniques for mining vehicles, such as model predictive control or sliding mode control, to benchmark the effectiveness of the elastic FA optimized filter adaptive PID controller against existing approaches.

Answer 5. Thank you for your valuable suggestions on this section. We will modify and improve this section based on your suggestions, and supplement the shortcomings of open-pit mine vehicle control technology (taking model predictive control and sliding mode control as examples), as well as the unsatisfactory results of simulation using them, which further highlights the advantages of elastic FA optimization filter adaptive PID control and the necessity of research.

REVIEW OF SECTION 4.3:

The authors present the verification of the simulation model for the KJCJ-4 mining concrete mixing and transport tank truck by comparing the simulation results with actual vehicle test data under a single lane shifting condition. They assess the accuracy and reliability of the established simulation model by examining the lateral acceleration curves obtained from the simulation and the actual vehicle test.

Verification Scenario:

The authors consider a single lane shifting condition with a filling rate of 1.5 and a vehicle speed of 50 km/h to verify the simulation model.

Areas requiring further clarification:

1. Justification for chosen scenario: The authors should provide more justification for choosing the single lane shifting condition and the specific filling rate and vehicle speed, discussing their relevance to the typical operating range of the mining concrete mixing and transport tank truck.

Answer 1. Thank you for your valuable comments on this section. We will modify and improve this section based on your comments. Due to the length of the article, we will elaborate and interpret it in detail as follows.

In the mining area, concrete mixer transport tank trucks often need to travel on narrow mine tunnels or single lanes. Selecting a single lane shift condition can simulate this actual operating environment. In order to ensure safe and efficient transportation management, vehicles usually drive along designated lanes and routes. The single lane shift condition can help evaluate the vehicle control performance under such constraints. Compared with multi-lane or complex routes, the single lane shift condition is easier to model and simulate, and can focus more on the speed control and stability evaluation of the vehicle.

In actual operation, concrete mixer transport tank trucks are usually not filled to avoid rollover or other safety problems due to unstable center of gravity during transportation. A high filling rate may increase the center of gravity height of the tank truck and affect driving stability. Selecting a moderate filling rate can better simulate the common loading conditions in actual operation and ensure the stability and safety evaluation of the simulation results. Selecting a filling rate of 1.5 can represent a more common loading situation. The shaking of concrete inside the tanker can reflect the fluid dynamics phenomenon that may occur during actual transportation, providing a basis for the robustness and adaptability test of the control strategy.

Roads in mining areas usually have strict speed limits to ensure safety and reduce accidents. Selecting 50 km/h as the vehicle speed can meet the common speed limit requirements in mining areas. In addition, while ensuring safety, increasing the vehicle speed can improve transportation efficiency. Selecting this speed can balance safety and efficiency and provide a typical speed reference in actual operation for simulation. Different vehicle speeds affect the dynamic characteristics and control requirements of the vehicle. Selecting this speed can test the performance of the controller at higher speeds and evaluate its applicability and reliability in efficient transportation.

The operating speed of mining concrete mixing tank trucks is usually limited by road conditions, vehicle loads and safety regulations in mining areas. 50 km/h is a more reasonable upper limit that can ensure transportation efficiency without excessively increasing safety risks. The setting of a filling rate of 1.5 reflects the common loading conditions in actual operations in mining areas, helps simulate the impact of concrete sloshing on vehicle stability, and provides actual data support for the optimization of control strategies. The single lane shift condition simulates the typical scenario of single-lane driving in mining areas, and can truly reflect the control requirements and performance of vehicles under narrow road conditions. By selecting a single lane shift condition, a filling rate of 1.5, and a vehicle speed of 50, the actual operating conditions of mining concrete mixing and transport tank trucks can be accurately reproduced in the simulation. This choice not only improves the reliability of the simulation results, but also provides a valuable reference for the optimization and evaluation of control strategies.

Lateral Acceleration Comparison (Figures 11(a) and 11(b)):

The results show that the simulation curve and the test curve exhibit good consistency in both the change trend and the range of lateral acceleration values, with a relative error within 7.2%.

Areas requiring further analysis:

1. Quantitative metrics for agreement: The authors should provide more quantitative metrics, such as the root mean square error (RMSE) or the coefficient of determination (R^2), to assess the agreement between the simulation and the test data objectively.

Answer 1. Thank you for your valuable comments on this section. We will modify and improve this section based on your opinions, and add more data that can prove the consistency between the evaluation simulation and test data, such as: root mean square error and relative error.

The root mean square error is a measure of the difference between the predicted value and the actual observed value, reflecting the size of the model's prediction error. The unit of RMSE is the same as the unit of the original data, which is suitable for scenarios where you need to know the average error between the predicted value and the actual value. Root mean square error is more sensitive to larger errors because the error is amplified by the square, which can highlight the impact of larger errors on model performance. Because it includes a squaring operation, the root mean square error is sensitive to outliers and may be significantly affected by outliers. It is suitable for scenarios that have strict requirements on prediction errors, such as data analysis of precision instruments, weather prediction, etc. Since RMSE is sensitive to large errors, it can highlight the shortcomings of the model in these situations.

Relative error is a measure of the percentage of forecast error relative to the actual value, reflecting the proportion of error in the actual value. Relative error is usually used in scenarios where it is necessary to compare the error sizes of data at different scales or different units. Relative error can eliminate the impact of data dimensions, allowing data at different scales to be compared. In addition, it also provides an intuitive percentage form, making the results easy to understand and interpret. When the actual value approaches zero, the relative error becomes very large and even tends to infinity, possibly leading to misleading results. It is suitable for errors that require comparison of data on different scales or different units, such as economic data analysis, data comparison of different measurement units, etc. The percentage form of the relative error makes it more interpretable in these scenarios.

Fluid Sloshing Interference (Figure 11(b)):

The results demonstrate that the simulation model accurately reproduces the lateral acceleration profile under fluid sloshing interference, with good agreement in both the change trend and the range of values.

Areas requiring further clarification:

1. Modeling of fluid sloshing interference: The authors should provide more details on how the fluid sloshing interference is modeled in the simulation and how it is introduced in the actual vehicle test.

Answer 1. Thank you for your valuable comments on this section. We will modify and improve this section based on your comments, and supplement the details of modeling fluid sloshing disturbance in simulation and how to introduce it in actual vehicle testing.

Fluid sloshing is usually modeled as the movement of a liquid with a free surface in a container, which may cause a series of dynamic problems, especially the fluctuation of lateral acceleration, which will affect the stability and safety of the vehicle. For a more accurate simulation, we use CFD software [48] to simulate the movement of liquid in the tank truck, set boundary conditions (such as the geometry of the tank truck, the physical properties of the liquid) and perform dynamic simulation. Finally, the CFD simulation results are imported into Simulink for joint simulation. As shown in Figure 11(b), through the above simulation steps, fluid sloshing disturbance is introduced into the lateral acceleration curve of the mining concrete mixing tank truck.

The specific steps of the simulation are introduced. By establishing a fluid sloshing disturbance model in Simulink and conducting actual vehicle tests, we can gain a deep understanding of the impact of fluid sloshing on the mining concrete mixing tank truck, and improve the vehicle's operating safety and stability by improving the control strategy. Sensitivity analysis and data validation can help further optimize the simulation model to make it closer to actual working conditions.

2. Impact on stability and safety: The authors should discuss the potential impact of fluid sloshing on the stability and safety of the mining concrete mixing tank truck and highlight the importance of accurately capturing these effects in the simulation model.

Answer 2. Thank you for your valuable comments on this section. We will modify and improve this section based on your comments, supplement the potential impact of fluid sloshing on the stability and safety of mining concrete mixer trucks, and emphasize the importance of accurately capturing these effects in the simulation model.

Fluid sloshing has important potential impacts on the stability and safety of mining concrete mixer trucks, covering multiple aspects such as lateral acceleration fluctuations, vehicle stability, driver control difficulty, structural fatigue, and material quality uniformity. Accurately capturing these effects in the simulation model is essential for optimizing vehicle design, developing effective control strategies, improving the effectiveness of driver training and assistance systems, and conducting safety assessments and cost-benefit analyses. Fluid sloshing can cause lateral acceleration fluctuations in tank trucks during driving, especially when turning and making sudden stops and turns. Fluctuating lateral acceleration increases the risk of vehicle loss of control and rollover. Fluid sloshing can also affect the center of gravity position and motion characteristics of the vehicle, thereby affecting the stability of the vehicle. The sloshing liquid mass can cause the vehicle to have unstable motion patterns, such as swinging and tilting. It also increases the difficulty for the driver to control the vehicle, especially in complex terrain or in bad weather conditions. Fluctuating lateral forces exert additional torque on the steering wheel, making the vehicle more difficult to control.

Detailed modeling and simulation can help fully understand and solve these complex dynamic problems, thereby improving the overall performance and safety of mining concrete mixer trucks.

Criticism and Suggestions:

1. Justification for verification scenario selection: The authors should provide more justification for the selection of the single lane shifting condition and the specific filling rate and vehicle speed used for verification, discussing their representativeness and relevance to the typical operating range of the mining concrete mixing and transport tank truck.

Answer 1. Thank you for your valuable comments on this section. We will modify and improve this section based on your comments. Due to the length of the article, we will elaborate and interpret it in detail as follows.

In the mining area, concrete mixer transport tank trucks often need to travel on narrow mine tunnels or single lanes. Selecting a single lane shift condition can simulate this actual operating environment. In order to ensure safe and efficient transportation management, vehicles usually drive along designated lanes and routes. The single lane shift condition can help evaluate the vehicle control performance under such constraints. Compared with multi-lane or complex routes, the single lane shift condition is easier to model and simulate, and can focus more on the speed control and stability evaluation of the vehicle.

In actual operation, concrete mixer transport tank trucks are usually not filled to avoid rollover or other safety problems due to unstable center of gravity during transportation. A high filling rate may increase the center of gravity height of the tank truck and affect driving stability. Selecting a moderate filling rate can better simulate the common loading conditions in actual operation and ensure the stability and safety evaluation of the simulation results. Selecting a filling rate of 1.5 can represent a more common loading situation. The shaking of concrete inside the tanker can reflect the fluid dynamics phenomenon that may occur during actual transportation, providing a basis for the robustness and adaptability test of the control strategy.

Roads in mining areas usually have strict speed limits to ensure safety and reduce accidents. Selecting 50 km/h as the vehicle speed can meet the common speed limit requirements in mining areas. In addition, while ensuring safety, increasing the vehicle speed can improve transportation efficiency. Selecting this speed can balance safety and efficiency and provide a typical speed reference in actual operation for simulation. Different vehicle speeds affect the dynamic characteristics and control requirements of the vehicle. Selecting this speed can test the performance of the controller at higher speeds and evaluate its applicability and reliability in efficient transportation.

The operating speed of mining concrete mixing tank trucks is usually limited by road conditions, vehicle loads and safety regulations in mining areas. 50 km/h is a more reasonable upper limit that can ensure transportation efficiency without excessively increasing safety risks. The setting of a filling rate of 1.5 reflects the common loading conditions in actual operations in mining areas, helps simulate the impact of concrete sloshing on vehicle stability, and provides actual data support for the optimization of control strategies. The single lane shift condition simulates the typical scenario of single-lane driving in mining areas, and can truly reflect the control requirements and performance of vehicles under narrow road conditions. By selecting a single lane shift condition, a filling rate of 1.5, and a vehicle speed of 50, the actual operating conditions of mining concrete mixing and transport tank trucks can be accurately reproduced in the simulation. This choice not only improves the reliability of the simulation results, but also provides a valuable reference for the optimization and evaluation of control strategies.

2. Quantitative metrics for agreement: The authors should include more quantitative metrics, such as the root mean square error (RMSE) or the coefficient of determination (R^2), to assess the agreement between the simulation and the test data objectively and provide a standardized measure of the simulation model's accuracy.

Answer 2. Thank you for your valuable comments on this section. We will modify and improve this section based on your opinions, and add more data that can prove the consistency between the evaluation simulation and test data, such as: root mean square error and relative error.

The root mean square error is a measure of the difference between the predicted value and the actual observed value, reflecting the size of the model's prediction error. The unit of RMSE is the same as the unit of the original data, which is suitable for scenarios where you need to know the average error between the predicted value and the actual value. Root mean square error is more sensitive to larger errors because the error is amplified by the square, which can highlight the impact of larger errors on model performance. Because it includes a squaring operation, the root mean square error is sensitive to outliers and may be significantly affected by outliers. It is suitable for scenarios that have strict requirements on prediction errors, such as data analysis of precision instruments, weather prediction, etc. Since RMSE is sensitive to large errors, it can highlight the shortcomings of the model in these situations.

Relative error is a measure of the percentage of forecast error relative to the actual value, reflecting the proportion of error in the actual value. Relative error is usually used in scenarios where it is necessary to compare the error sizes of data at different scales or different units. Relative error can eliminate the impact of data dimensions, allowing data at different scales to be compared. In addition, it also provides an intuitive percentage form, making the results easy to understand and interpret. When the actual value approaches zero, the relative error becomes very large and even tends to infinity, possibly leading to misleading results. It is suitable for errors that require comparison of data on different scales or different units, such as economic data analysis, data comparison of different measurement units, etc. The percentage form of the relative error makes it more interpretable in these scenarios.

3. Verification under diverse operating conditions: The authors should consider verifying the simulation model under a wider range of operating conditions, such as different filling rates, vehicle speeds, and road profiles, to demonstrate the robustness and versatility of the simulation model in capturing the vehicle dynamics under diverse scenarios.

Answer 3. Thank you for your valuable comments on this section. We will modify and improve this section based on your comments. Due to the length of the article, we will elaborate and interpret it in detail as follows.

Simulate the dynamic behavior of the vehicle at different filling rates. Evaluate the impact of different filling rates on fluid sloshing, vehicle center of gravity position and its stability. Use the simulation model to perform multiple simulations, compare the differences between the actual test data and the simulation results, and adjust the model parameters to improve accuracy. Then analyze the impact of different vehicle speeds on fluid sloshing and vehicle lateral acceleration, especially in high-speed cornering and emergency braking. Construct various road profiles in the simulation environment, simulate the driving behavior of the vehicle on these roads, and compare the actual road test data and simulation results. Establish an initial simulation model based on the physical parameters of the vehicle (such as mass, center of gravity position, tire characteristics, etc.). Simulate the dynamic behavior of the vehicle under the same operating conditions at different filling rates, and record key indicators such as lateral acceleration and tilt angle. Based on the comparative analysis results, further adjust and optimize the simulation model parameters to improve the model's prediction ability under different operating conditions.

Through simulation and actual testing under different filling rates, vehicle speeds and road profiles, the robustness and versatility of the simulation model in capturing the dynamic behavior of the mining concrete mixer tanker was verified. Accurately capturing the dynamic characteristics under these different operating conditions is crucial to optimizing vehicle design, improving the effectiveness of control strategies, and ensuring the stability and safety of the vehicle in various operating scenarios. By continuously adjusting and optimizing the simulation model, it is possible to better predict and respond to complex dynamic problems in actual operations and improve the overall performance of the mining concrete mixer tanker.

4. Experimental setup and data acquisition details: The authors should provide more details on the experimental setup and the data acquisition system used for the actual vehicle test, discussing the sensors used, the sampling rate, and any signal processing techniques applied to ensure the reliability and consistency of the test data.

Answer 4. Thank you for your valuable comments on this section. We will modify and improve this section based on your comments, add more details of the experimental setup and data acquisition system for actual vehicle testing, discuss the sensors used, sampling rates, and any signal processing techniques applied to ensure the reliability and consistency of test data.

Select a vehicle that is consistent with the simulation model in all parameters, including vehicle mass, liquid filling rate, tire characteristics, etc. Select a test site that can simulate various road conditions (such as flat roads, ramps, and rough roads). Ensure that the test site is safe and equipped with necessary safety equipment.

Use ADXL345 sensors, L3GD20 gyroscopes, GPS modules, HC-SR04 liquid level sensors, Strain Gauge force sensors, etc. in the data acquisition system. The sensors should be installed in key locations of the vehicle, such as near the center of gravity of the vehicle body, inside the liquid tank, and near the wheels. The sensors must be firmly fixed to avoid movement or loosening during the test. Choose an appropriate sampling rate based on the frequency characteristics of the vehicle's dynamic behavior. The sampling rate of acceleration and angular velocity should be above 100 Hz to capture rapidly changing dynamic behaviors. Ensure that data from all sensors are collected synchronously so that accurate time alignment can be performed for subsequent analysis. Use a high-performance data acquisition system that can process multi-channel high-frequency data simultaneously. Then conduct actual vehicle tests to evaluate the stability and fluid sloshing characteristics of the vehicle when driving on a flat road.

Through detailed experimental setup and data acquisition system design, high-quality data can be obtained in actual vehicle tests. Using appropriate sensors, sampling rates, and signal processing techniques, the dynamic behavior of mining concrete mixer transport tank trucks under different operating conditions can be accurately captured. These data are critical to verifying the accuracy and robustness of the simulation model, thereby helping to optimize vehicle design and control strategies and improve the overall performance and safety of the vehicle.

5. Analysis of discrepancy sources: The authors should analyze the sources of discrepancy between the simulation and the test results, such as modeling assumptions, parameter uncertainties, and measurement errors, discussing their potential impact on the simulation model's accuracy and proposing strategies for minimizing their effects.

Answer 5. Thank you for your valuable comments on this section. We will modify and improve this section based on your comments, add more details of the experimental setup and data acquisition system for actual vehicle testing, discuss the sensors used, sampling rates, and any signal processing techniques applied to ensure the reliability and consistency of test data.

When simulating and testing mining concrete mixer transport tankers, the differences between the results may arise from a variety of factors, including modeling assumptions, parameter uncertainties, and measurement errors. These differences may affect the accuracy of the simulation model, so detailed analysis and measures need to be taken to minimize their impact.

In order to simplify the calculation, some simplifying assumptions may be made in the model about the dynamic behavior of the vehicle, liquid sloshing, and road conditions. Simplified assumptions may cause the model to fail to accurately capture the complex dynamic behavior of the actual vehicle. The linearization process may ignore nonlinear effects, resulting in deviations in the simulation results. The physical properties of the materials of vehicle components (such as elastic modulus, damping coefficient) may have uncertainties. The actual vehicle configuration (such as mass distribution, tire characteristics) may also differ from the model settings. The environmental conditions in the actual test (such as temperature, humidity, wind speed) may be different from the standard environmental conditions assumed by the model. The measurement accuracy and resolution of the sensor may affect the accuracy of the data. Noise and delays in the data acquisition system may lead to inaccurate measurement data.

In order to minimize the difference between simulation and actual test results, it is necessary to comprehensively consider modeling assumptions, parameter uncertainties, and measurement errors, and adopt corresponding strategies for optimization and calibration. Specifically, it includes increasing model complexity, performing parameter identification and uncertainty analysis, using high-precision sensors and advanced data processing technology, and performing multiple repeated tests. These measures help improve the accuracy and reliability of simulation models, thereby better guiding the design and control strategy optimization of mining concrete mixing and transport tank trucks. Errors may be introduced by filtering and calibration methods in the data processing process.

Your critical review of the "Conclusion and Outlook" section provides a comprehensive analysis of the main contributions, findings, and potential improvements in the paper. You have summarized the key points discussed by the authors, identified missing elements in the outlook, and highlighted inconsistencies with the main idea of the paper. Let's organize your review into a structured format to enhance clarity and readability.

REVIEW OF SECTION 5:

Missing Elements in the Outlook:

The authors do not explicitly provide an outlook or future research directions. They could have discussed potential extensions of their work, such as:

1. Experimental validation of the proposed control algorithm on a real concrete mixing tank truck.

2. Integration of the control algorithm with advanced sensing and monitoring technologies.

3. Investigation of the long-term durability and reliability of the hydrostatic drive system components under the proposed control scheme.

4. Extension of the research to other types of heavy-duty vehicles used in open-pit mines.

Answer: Thank you for your four comments on the outlook section. We have made corresponding supplements and improvements based on your comments. This time, the conclusion and outlook are explained separately, which can more clearly explain the content of this article. The outlook is a plan for the future. The elastic firefly algorithm optimized filter adaptive PID control strategy proposed in this paper provides an effective solution for the speed control of mining concrete mixer transport tank trucks. However, the actual application of the control algorithm still needs further verification and optimization. Through experimental tests on real vehicles, combining advanced sensing and monitoring technologies, studying the long-term durability of the hydrostatic drive system, and expanding the research to other types of heavy vehicles, we hope to comprehensively improve the performance and reliability of the control system and provide a solid guarantee for the efficient and safe operation of open-pit mine vehicles.

4.2 Outlook

(1) In order to verify the effectiveness and performance of the control algorithm proposed in this paper under actual working conditions, we plan to conduct experimental tests on real mining concrete mixer tank trucks. These tests will cover a variety of operating conditions, including different filling rates, vehicle speeds, and road profiles to evaluate the robustness and adaptability of the control algorithm in real environments. By comparing the experimental results with the simulation results, we will further optimize the control algorithm to ensure its reliability in actual applications.

(2) The performance of the control algorithm can be further improved by combining advanced sensing and monitoring technologies. For example, the use of high-precision accelerometers, gyroscopes, liquid level sensors, and force sensors can monitor the dynamic behavior of the vehicle and the sloshing of the liquid in real time. By integrating with the Internet of Things technology, remote monitoring and data analysis can be achieved, thereby improving the operational safety and efficiency of the vehicle. Future research will explore how to effectively combine these advanced technologies with the control algorithm proposed in this paper to build an intelligent and efficient control system.

(3) The hydrostatic drive system is an important component of the mining concrete mixer tank truck, and its long-term durability and reliability are crucial to the performance of the entire vehicle. Under the proposed control scheme, we will study the long-term use of the components of the hydrostatic drive system, including the wear and failure of key components such as the hydraulic pump, hydraulic motor, and control valve. Through long-term experimental testing and data analysis, we will evaluate the impact of the control algorithm on the durability and reliability of the hydrostatic drive system and propose corresponding improvement measures.

(4) Although the focus of this paper is on mining concrete mixer transport tank trucks, the proposed control algorithm has a wide range of applicability. Future research will be extended to other types of heavy vehicles used in open-pit mines, such as mining dump trucks, excavators, and loaders. These heavy vehicles also face complex dynamic behaviors and harsh operating conditions in the operating environment of open-pit mines. Through verification and optimization on different types of heavy vehicles, we will further improve the versatility and robustness of the control algorithm and provide strong support for the intelligent and automated operation of open-pit mines.

Inconsistencies and Problems:

1. Lack of a clear and concise summary of the main objectives and methodologies.

2. Insufficient explanation of how the ground mechanics and hydrostatic drive system models support the development and evaluation of the proposed control algorithm.

3. Missing explicit link between the liquid sloshing dynamics findings and the adaptive control algorithm's performance.

4. Absence of a clear statement of the practical implications and potential benefits of the proposed algorithm for open-pit mining operations.

Recommendations:

1. Include a brief restatement of the research problem and the proposed solution approach at the beginning of the conclusion section to help readers recall the main idea of the paper.

2. Explain how the ground mechanics and hydrostatic drive system models support the development and evaluation of the proposed control algorithm.

3. Highlight how the proposed algorithm helps mitigate the adverse effects of liquid sloshing on vehicle stability and safety.

4. Emphasize the practical implications and potential benefits of the proposed adaptive hydraulic drive control algorithm for open-pit mining operations.

5. Provide a clear outlook on future research directions, addressing the identified missing elements.

Answer: Thank you for your valuable comments on this section. We will revise and improve this section based on all your comments above. We will add more explanations and briefly restate the research questions and proposed solutions at the beginning of the conclusion section. The content supported by the model for the proposed algorithm is included, and a fourth article is added at the end of the conclusion to emphasize how the proposed algorithm can help reduce the negative impact of liquid sloshing on vehicle stability and safety and the practical significance of the algorithm for open-pit mining operations. and potential benefits. Finally, the content of the outlook part is re-elaborated to make the "Conclusion and Outlook" part more clear-cut so that readers can better understand its meaning.

(4) The proposal of the optimized particle filter PID adaptive control method of the elastic firefly algorithm can effectively prevent the vehicle from being affected by complex road excitations by significantly reducing the speed error and improving the steady-state accuracy compared with the traditional algorithm. The body coupling phenomenon caused by the liquid sloshing reduces the negative impact of liquid sloshing on vehicle stability and safety, which can greatly improve the efficiency, equipment stability and safety of open-pit mine operations. Potential benefits include improved operating efficiency, enhanced equipment stability, improved operating environment and reduced operating costs. This advanced control method brings a more reliable, efficient and economical solution to open pit mining operations. At the same time, it will further promote the efficient, safe and sustainable development of open-pit mining operations by reducing costs, improving safety, protecting the environment and supporting data-driven decision-making.

By incorporating these recommendations, the authors can enhance the clarity, coherence, and impact of the "Conclusion and Outlook" section, ensuring that it effectively summarizes the main contributions and findings of the research while maintaining consistency with the main idea of the paper.
